# Inhibition of the autophagic protein ULK1 attenuates axonal degeneration in vitro and in vivo, enhances translation, and modulates splicing

**DOI:** 10.1038/s41418-020-0543-y

**Published:** 2020-04-27

**Authors:** Björn Friedhelm Vahsen, Vinicius Toledo Ribas, Jonas Sundermeyer, Alexander Boecker, Vivian Dambeck, Christof Lenz, Orr Shomroni, Lucas Caldi Gomes, Lars Tatenhorst, Elisabeth Barski, Anna-Elisa Roser, Uwe Michel, Henning Urlaub, Gabriela Salinas, Mathias Bähr, Jan Christoph Koch, Paul Lingor

**Affiliations:** 1 Department of Neurology, University Medical Center Göttingen, Robert-Koch-Str. 40, 37075 Göttingen, Germany; 2 Department of Morphology, Universidade Federal de Minas Gerais, Av. Pres. Antônio Carlos, 6627, Pampulha, Belo Horizonte, MG 31270-901 Brazil; 3 Department of Physiology, Perelman School of Medicine, University of Pennsylvania, 630 Clinical Research Building, 415 Curie Boulevard, Philadelphia, PA 19104 USA; 4 Center for Biostructural Imaging of Neurodegeneration (BIN), University Medical Center Göttingen, Von-Siebold-Str. 3a, 37075 Göttingen, Germany; 5 DFG Cluster of Excellence Nanoscale Microscopy and Molecular Physiology of the Brain (CNMPB), University Medical Center Göttingen, Robert-Koch-Str. 40, 37075 Göttingen, Germany; 6 Institute of Clinical Chemistry, University Medical Center Göttingen, Robert-Koch-Str. 40, 37075 Göttingen, Germany; 7 Bioanalytical Mass Spectrometry, Max Planck Institute for Biophysical Chemistry, Am Faßberg 11, 37077 Göttingen, Germany; 8 NGS-Integrative Genomics Core Unit (NIG), Institute of Human Genetics, University Medical Center Göttingen, Justus-von-Liebig-Weg 11, 37077 Göttingen, Germany; 9 Department of Neurology, Rechts der Isar Hospital, Technical University Munich, Ismaninger Str. 22, 81675 Munich, Germany

**Keywords:** Neuroscience, Neurological disorders, Experimental models of disease, Translational research

## Abstract

Axonal degeneration is a key and early pathological feature in traumatic and neurodegenerative disorders of the CNS. Following a focal lesion to axons, extended axonal disintegration by acute axonal degeneration (AAD) occurs within several hours. During AAD, the accumulation of autophagic proteins including Unc-51 like autophagy activating kinase 1 (ULK1) has been demonstrated, but its role is incompletely understood. Here, we study the effect of ULK1 inhibition in different models of lesion-induced axonal degeneration in vitro and in vivo. Overexpression of a dominant negative of ULK1 (ULK1.DN) in primary rat cortical neurons attenuates axotomy-induced AAD in vitro. Both ULK1.DN and the ULK1 inhibitor SBI-0206965 protect against AAD after rat optic nerve crush in vivo. ULK1.DN additionally attenuates long-term axonal degeneration after rat spinal cord injury in vivo. Mechanistically, ULK1.DN decreases autophagy and leads to an mTOR-mediated increase in translational proteins. Consistently, treatment with SBI-0206965 results in enhanced mTOR activation. ULK1.DN additionally modulates the differential splicing of the degeneration-associated genes *Kif1b* and *Ddit3*. These findings uncover ULK1 as an important mediator of axonal degeneration in vitro and in vivo, and elucidate its function in splicing, defining it as a putative therapeutic target.

## Introduction

In both traumatic and neurodegenerative disorders of the CNS, axonal degeneration occurs early in the disease course and frequently precedes neuronal cell death [1–4]. The regenerative capacity of CNS axons, however, is very limited [5]. Axonal pathology thus often leads to irreversible neurological deficits causing progressive disability. A better understanding of the underlying mechanisms raises the hope to develop novel therapeutic strategies [1, 6].

After focal traumatic injury to axons, sudden axonal disintegration named acute axonal degeneration (AAD) occurs within the first few minutes, extending for up to 500 µm on both sides of the lesion within several hours [7, 8]. During AAD, one of the first steps is rapid intra-axonal calcium influx leading to the activation of calpain proteases and macroautophagy (here, autophagy) [7, 8]. Inhibition of calcium channels and calpain successfully attenuate AAD [8–10]. The role of autophagy in axonal degeneration, however, remains not fully understood.

After optic nerve crush (ONC), application of the autophagy inhibitor 3-methyladenine (3-MA) led to the partial inhibition of AAD [11]. Recently, we demonstrated increased levels of autophagic proteins, including Unc-51 like autophagy activating kinase 1 (ULK1), early in degenerating axons after spinal cord injury (SCI) [12]. ULK1 is a key protein involved in the initiation of autophagy and has previously been shown to be implicated in neurite outgrowth in a kinase screen [13, 14]. Whether a modulation of ULK1 could be beneficial in blocking lesion-induced axonal degeneration has not been evaluated so far.

Therefore, in this study, we overexpress a dominant negative of ULK1 (ULK1.DN) [15] and investigate its effect on axonal degeneration in different models of axonal lesion in vitro and in vivo. In a translational approach, we additionally administer the small-molecule ULK1 inhibitor SBI-0206965 [16].

## Materials and methods

### Cloning and production of adeno-associated viral vectors

Cloning and production of AAV.ULK1.DN (corresponding to amino acids 829–1051 of ULK1, connected to an N-terminal myc-tag [15]) and AAV.CTRL was performed as reported previously [17]. In addition, AAV.EGFP [18, 19] was used in some in vivo experiments.

### Neuronal cell culture, viral transduction, and toxicity assay

Primary rat cortical neurons were prepared from embryonic day 18 rats as described before [10] and cultured in cortex medium composed of serum-free neurobasal medium supplemented with B-27, penicillin/streptomycin/neomycin, l-glutamine (all Thermo Fisher Scientific, Waltham, MA, USA), and transferrin (AppliChem, Darmstadt, Germany) at 37 °C and 5% CO_2_. On day in vitro (DIV) 1, cells were transduced with AAV.ULK1.DN and AAV.CTRL. Rapamycin (750 nM), staurosporine (30–300 nM), and SBI-0206965 (5 μM) or DMSO as control (all Sigma-Aldrich, St. Louis, MO, USA) were applied in selected conditions. For some experiments, medium samples were used for a bioluminescence-based toxicity assay (ToxiLight™, Lonza, Basel, Switzerland). Details of cell culture experiments can be found in the Supplementary Information (SI).

### Cell lysis and western blot analysis

Cortical neurons transduced with AAV.ULK1.DN and AAV.CTRL were lysed in lysis buffer composed of 0.5% (v/v) Nonidet P-40, 20 mM HEPES, 300 mM NaCl (all AppliChem), 5 mM EDTA, 1 mM dithiothreitol (both Sigma-Aldrich), plus protease inhibitor (cOmplete™), and phosphatase inhibitor (PhosSTOP™, both Roche, Basel, Switzerland). The protein content of each sample was determined using Pierce^TM^ bicinchoninic acid assay kit (Thermo Fisher Scientific).

Western blot analysis was performed using the following primary antibodies: rabbit anti-thousand and one amino acid protein kinase 1 (anti-TAOK1, 1:1000, ab52097, Abcam, Cambridge, UK), mouse anti-microtubule-associated protein 1 light chain 3B (anti-LC3, 1:1000, 5F10, nanoTools, Teningen, Germany), mouse anti-β-tubulin (1:80,000, T4026), rabbit anti-p62 (1:2000, P0067), rabbit anti-ULK1 (1:1000, A7481, all Sigma-Aldrich), rabbit anti-ULK2 (1:500, MBS9610206, MyBioSource, San Diego, CA, USA), mouse anti-eukaryotic translation initiation factor 4E binding protein 1 (anti-4E-BP1, 1:200, AHO1382, Thermo Fisher Scientific), rabbit anti-mammalian target of rapamycin (anti-mTOR, 1:1000, 04-385, Merck, Darmstadt, Germany) or mouse anti-mTOR (1:1000, 4517, Cell Signaling Technology, Danvers, MA, USA), rabbit anti-autophagy-related protein 5 (anti-ATG5, 1:500, AP1812b, Abgent, San Diego, CA, USA), mouse anti-glyceraldehyde 3-phosphate dehydrogenase (anti-GAPDH, 1:5000, 5G4, Hytest Ltd., Turku, Finland), rabbit anti-autophagy-related protein 7 (anti-ATG7, 1:400, 2631S), rabbit anti-caspase 3 (1:500, 9662), rabbit anti-myc-tag (1:1000, 2272), rabbit anti-AMP-activated protein kinase alpha (anti-AMPKα, 1:1000, 2532) or mouse anti-AMPKα (1:1000, 2793), rabbit anti-p-AMPKα (1:1000, 2535), rabbit anti-p-mTOR (1:1000, 5536), mouse anti-S6 ribosomal protein (anti-S6, 1;1000, 2317), rabbit anti-phospho-S6 (anti-p-S6, 1:1000, 2211), rabbit anti-phospho-4E-BP1 (anti-p-4E-BP1, 1:250, 2855), rabbit anti-p21 (RAC1) activated kinase 2 (anti-PAK2, 1:1000, 2608S, all Cell Signaling Technology).

This was followed by incubation with the following corresponding horseradish peroxidase (HRP)-coupled secondary antibodies: horse anti-mouse HRP (1:1000, 7076P2), goat anti-rabbit HRP (1:1000, 7074P2, both Cell Signaling Technology). Alternatively, the following fluorescent antibodies were used: donkey IRDye 680RD anti-rabbit (1:5000, 925-68073), donkey IRDye 680RD anti-mouse (1:5000, 926-68072), goat IRDye 800CW anti-rabbit (1:10,000, 926-32211), goat IRDye 800CW anti-mouse (1:10,000, 926-32210, all LI-COR-Biosciences, Lincoln, NE, USA).

Details of cell lysis and western blotting procedures can be found in the SI.

### RNA isolation, RNA-seq, differential exon, and gene expression analyses

Cortical neurons transduced with AAV.ULK1.DN and AAV.CTRL were lysed with TRI-reagent (Sigma-Aldrich) for total RNA extraction.

Quality and integrity of RNA was assessed with the Fragment Analyzer (Advanced Analytical, Santa Clara, CA, USA) by using the standard sensitivity RNA Analysis Kit (DNF-471, Advanced Analytical). All samples selected for sequencing exhibited an RNA integrity number over 8. RNA-Seq libraries were produced using 500 ng total RNA and the TruSeq RNA Library Preparation Kit v2 (set A; 48 samples, 12 indexes, Illumina, San Diego, CA; Cat. no. RS-122-2001). Specifically, after first optimization of the ligation step by diluting the adapters concentration to increase ligation efficiency (>94%), the number of PCR cycles (ten cycles) was reduced to avoid PCR duplication artifacts as well as primer dimers in the final library product. Libraries were prepared on the Biomek FXP workstation (Beckman Coulter, Brea, CA, USA). For accurate quantitation of cDNA libraries, a fluorometric based system (QuantiFluor™ dsDNA System; Promega, Madison, WI, USA) was used. The size of final cDNA libraries was determined by using the dsDNA 905 Reagent Kit (Fragment Analyzer, Advanced Analytical) exhibiting a sizing of 300 bp on average. Libraries were pooled (six samples) and sequenced on the rapid mode of the Illumina HiSeq 2500 (PE; 2 × 250 bp; 40–50 Mio reads/sample). Details of RNA isolation, RNA-seq, and data processing can be found in the SI.

Visualization of differentially expressed exons was performed using Circos version 0.69 [20]. Functional annotation of genes with differential exon expression to Gene Ontology (GO) terms was performed in DAVID version 6.8 [21]. The functional annotation module was applied for GO biological process, molecular function, and cellular component terms using an EASE score of 0.1 and a minimum number of 2 counts.

### Proteomics analysis

Cortical neurons transduced with AAV.ULK1.DN and AAV.CTRL were lysed and the protein content of each sample was determined as described above. After precipitation with acetone, 50 µg of protein lysates per sample were separated on a 4–12% NuPAGE Novex Bis-Tris Minigel (Thermo Fisher Scientific). Following Coomassie staining, the protein areas were cut out, diced, and subjected to reduction with dithiothreitol, alkylation with iodoacetamide, and finally ON digestion with trypsin. Tryptic peptides were extracted from the gel, the solution dried in a Speedvac, and subjected to nanoLC-MS/MS as described previously [10, 22]. Details of mass spectrometric analysis and data processing are provided in the SI.

Functional annotation of differentially regulated proteins to GO terms was performed in DAVID version 6.8 [21]. The functional annotation module was applied for GO biological process and cellular component terms using an EASE score of 0.1 and a minimum number of 2 counts. For analysis of protein–protein interaction networks, STRING version 10.5 [23] was used with a minimum required interaction score of 0.4. The STRING database’s k-Means clustering tool was employed to group proteins with roles in similar processes into four clusters.

### Live imaging and quantification of axonal degeneration in microfluidic chambers in vitro

Microfluidic chambers were produced based on previously published protocols [10, 24, 25]. Cortical neurons were seeded into each chamber and transduced with AAV.ULK1.DN and AAV.CTRL. On DIV 7–9, axons reached the axonal compartment of the chambers. An axotomy was performed by applying air bubbles to the axonal compartment with gentle vacuum aspiration, leading to the induction of axonal degeneration. Axons were imaged in a microscope incubation system (DMI6000B, Leica, Wetzlar, Germany) equipped with Leica Application Suite software directly before and 5 min–6 h after axotomy. The number of bulb-like structures (diameter > 2 µm) was quantified in single axons within 100–400 µm proximal to the lesion site for all time points in a blinded fashion. The number of bulbs before axotomy was then subtracted from the bulb number at each time point to determine the number of newly formed bulbs [10]. Details of microfluidic chamber culture, live imaging, and quantification can be found in the SI.

### Animal experiments

All animal experiments were performed with the approval of the governmental authorities and according to the legislation of the local animal research council of the State of Lower Saxony (Braunschweig), Germany. Adult female wistar rats weighing 200–300 g were used for all in vivo experiments. The animals were randomly allocated to each group.

All procedures with AAV.ULK1.DN and AAV.CTRL (stereotactical injection, SCI, intravitreal virus injection, optic nerve live imaging, and crush) were performed under deep anesthesia with 10% ketamine (95 mg/kg body weight) and 2% xylazine (7 mg/kg body weight) injected intraperitoneally. All procedures with the ULK1 inhibitor SBI-0206965 (intravitreal injections, optic nerve live imaging) were performed under deep anesthesia with intraperitoneal injection of 10% ketamine (75 mg/kg body weight) and 1 mg/ml medetomidine (0.5 mg/kg body weight). Details of animal care are provided in the SI.

### Stereotactic viral injection into the red nucleus (RN), SCI, and histology

To transduce RN neurons and their axons in the rubrospinal tract (RST), stereotactical injections of AAV.ULK1.DN and AAV.CTRL into the left RN were performed according to a previously published protocol [26, 27]. Five weeks later, a SCI was performed as described before [12, 26]. Briefly, the animals were anesthetized as described above, the skin on the back was incised and the fat layer on the back muscles was separated. After splitting of the musculature, the spinal cord was exposed via dorsal laminectomy of the thoracic level 8 (Th8) vertebra. The dorsal right half of the spinal cord was transected at Th8 using a pair of microscissors at a depth of 1.25 mm, which resulted in a complete axotomy of the RST. Finally, back muscles and skin were sutured, and animals were allowed to recover from anesthesia. Seven days after SCI, the animals were perfused and prepared for cryosectioning as described before [26]. Spinal cord tissue was partitioned into a block around the lesion (1.5 cm length) and a 0.5 cm block at Th2 rostral to the lesion. The lesion block, including the lesion epicenter and the rostral and caudal regions, was cryosectioned horizontally (30 µm) at the level of the RST. Coronal cryosections of the spinal cord at Th2 level were prepared at a thickness of 30 µm.

Details of injections, surgery, and tissue preparation can be found in the SI.

### Quantification of axonal degeneration in the spinal cord

To quantify axonal degeneration, horizontal spinal cord sections containing the RST (60 µm apart) were imaged using an upright fluorescence microscope (Axioplan, Zeiss, Oberkochen, Germany) equipped with AxioVision Software (Zeiss). Axonal degeneration in the rostral region was quantified at defined distances (500–2000 µm) from the lesion epicenter using a superimposed counting grid. We counted the number of intact (no signs of fragmentation visible) mCherry-positive axons per section in a blinded fashion. An axonal degeneration index was determined by normalization of the number of intact axons to the total number of mCherry-positive axons counted on coronal sections rostral to the lesion at Th2 in each animal. In the caudal region, Wallerian degeneration was quantified as the percentage of axons scored as degenerated (any sign of fragmentation visible) among all axons at 4 mm caudal to the lesion epicenter. Animals with low virus transduction (<250 axons/section), verified by counting the number of mCherry-positive axons in spinal cord coronal sections at Th2, were excluded.

### Quantification of lesion size in the spinal cord

For the quantification of lesion areas, spinal cord horizontal cryosections of the lesion site were stained with an antibody rabbit anti-glial fibrillary acidic protein (anti-GFAP, 1:300, Z0334, DAKO, Santa Clara, CA, USA), followed by secondary antibody goat anti-rabbit Alexa 488-labeled (1:500, 111-545-144, Dianova, Hamburg, Germany), counter-stained with DAPI (Sigma-Aldrich) and mounted in Mowiol (Hoechst, Frankfurt, Germany). To define the lesion area, sections containing the RST (60 µm apart) and immunostained with GFAP were imaged using an upright fluorescence microscope (Axioplan) equipped with AxioVision Software. Areas with little or no GFAP immunoreactivity were defined as the lesion [28] and measured using AxioVision software in a blinded fashion.

### Quantification of the number of autophagosomes in degenerating axons after SCI

To quantify the number of autophagosomes in degenerating axons after SCI, horizontal spinal cord sections were immunostained with an antibody goat anti-LC3 (1:50, sc-16756, Santa Cruz, Dallas, TX, USA) and developed with a secondary antibody donkey anti-goat Cy5-labeled (1:500, 705-175-147, Dianova). Using a confocal microscope (LSM710, Zeiss) equipped with ZEN software, images were taken at 500–1000 µm rostral to the lesion epicenter using an oil immersion 40× objective. The number of LC3 puncta was quantified per area of mCherry-positive degenerated axons in a blinded fashion using ImageJ software.

### Intravitreal injections, ONC, live imaging, and quantification of AAD in vivo

To evaluate AAD in vivo, rat ONC and live imaging were performed as described previously [29]. Twenty-one days before ONC, AAV.ULK1.DN and AAV.CTRL were injected intravitreally using a Hamilton syringe (701RN, 26s gauge, Hamilton, Reno, NV, USA) to transduce retinal ganglion cells. In additional experiments, intravitreal injections of AAV.EGFP were performed 14 days before ONC to label the axons of the optic nerve and SBI-0206965 (dissolved in DMSO to 20 mM stock solution, diluted in deionized H_2_O to 5 µM or 50 µM working concentration; APExBIO, Boston, MA, USA) or DMSO as control (AppliChem, 0.25% working solution) were injected intravitreally 2.5 h prior to ONC. In brief, the skin of the deeply anesthetized animal was incised close to the orbital rim, the orbital cavity was opened, and the lacrimal gland was moved to the front or partly removed. The superior rectus muscle was detached from its insertion point and the eye bulb was rotated laterally. The optic nerve was exposed by a longitudinal incision of the optic nerve sheath. Care was taken not to damage the central retinal artery. The crush injury was performed by tightly constricting a 10-0 polyamide suture (Johnson & Johnson, New Brunswick, NJ, USA) around the optic nerve at ~1 mm from the insertion of the optic nerve into the eye bulb for 30 s. The knot was left on the nerve for visualization of the crush site. Live imaging of AAD was performed using an Axio Examiner.Z1 microscope (Zeiss) equipped with a 40×/1.0 NA water immersion objective. Z-stack images were taken in the area of 400–500 µm proximal to the crush site 5–360 min after crush using ZEN software.

To quantify axonal degeneration, representative pictures were generated as 2D projections from the original Z-stack images using ZEN software. Image analysis was performed with ImageJ in a blinded fashion. The Axonal Integrity Ratio (AIR) was calculated by dividing the sum lengths of the remaining axon fragments by the original axon length for each axon at all time points [29].

Details of the surgical procedure, live imaging, and quantification are provided in the SI.

### Immunohistochemistry and quantification of LC3- and p62-puncta in the optic nerve

Optic nerves were fixed in 4% paraformaldehyde at 4 °C overnight and incubated in 30% sucrose for at least 24 h. Longitudinal cryosections of the optic nerve (16 µm thickness) were obtained and rehydrated in 0.05 M Tris for 15 min. Sections were stained with the following primary antibodies: rabbit anti-p62 (1:2000, P0067, Sigma-Aldrich), goat anti-LC3 (1:50, sc-16756, Santa Cruz), mouse anti-SMI32 (1:1000, SMI-32P, Covance, Princeton, NJ, USA). The following secondary antibodies were used: Alexa Fluor 546 donkey anti-rabbit (1:250), Alexa Fluor 647 donkey anti-mouse (1:250, both Thermo Fisher Scientific), Cy3 donkey anti-goat (1:250, Dianova). After counterstaining with DAPI, sections were embedded in DABCO mounting medium (Sigma-Aldrich).

Confocal microscopy was performed with a Leica TCS SP5 microscope (40×/1.25 NA oil immersion objective, ×5 digital zoom, sequential scanning). Images were taken at ~400 µm proximal and distal to the crush lesion. Intra axonal LC3- and p62-puncta were quantified with ImageJ software in a blinded fashion and normalized to the axon area of the image. Details of the quantification can be found in the SI.

### Data analysis

Samples sizes used in this study were similar to those routinely used in this field previously. Therefore, no statistical methods were used to additionally predetermine sample sizes. All experiments (cell culture, SCI, and ONC) are routinely performed in our lab. Statistical analyses were conducted using Prism 7 software (GraphPad Software, Inc., La Jolla, CA, USA). Data distribution was assumed to be normal and variances were assumed to be similar, without formal testing. One-sample *t*-test was used to test single groups. Comparisons of two groups were done by two-tailed unpaired *t*-test, multiple group comparisons by one-way analysis of variance (ANOVA) with Dunnett’s, Tukey’s, or Sidak’s post hoc test, and two-way repeated measurement ANOVA with Tukey’s or Sidak’s post hoc test. The statistical test and number of in vitro or in vivo experiments used for each analysis are indicated in each figure legend. Data are presented as single data points and means ± SEM. Differences were considered significant when *P* < 0.05 (*P < 0.05; **P < 0.01; ***P < 0.001; N.S. not significant). Final assembly and preparation of all figures for publication was done using CorelDRAW 2017 (Corel Corporation, Ottawa, Canada).

## Results

### AAV.ULK1.DN alters levels of autophagic proteins ULK1, LC3-II, and p62

**Fig. 1:**
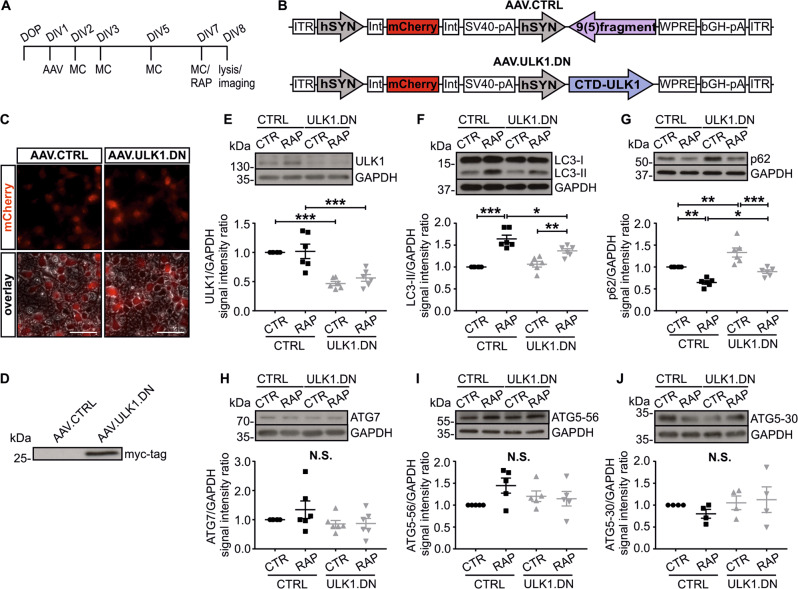
AAV.ULK1.DN-mediated overexpression of dominant-negative ULK1 in vitro alters the levels of the autophagic proteins ULK1, LC3-II, and p62. **a** Scheme of experimental setup. DOP day of preparation of embryonic day 18 (E18) rat cortical neurons. DIV day in vitro, AAV transduction with adeno-associated viral vectors, MC medium change, RAP addition of rapamycin (750 nM) 24 h before lysis. **b** Vector maps of the AAV used to express the mCherry fluorophore as well as the untranslated 9(5) fragment (AAV.CTRL) or the C-terminal domain (CTD) of ULK1, which has dominant-negative properties (AAV.ULK1.DN), both of them expressed under the control of a human synapsin promoter (hSYN). ITR AAV-2 inverted terminal repeat, Int intron, SV40-pA SV40-polyadenylation site, WPRE Woodchuck hepatitis virus posttranscriptional regulatory element. bGH-pA bovine growth hormone-polyadenylation site. **c** Photomicrographs (DIV 8) of E18 rat cortical neurons transduced with AAV.CTRL or AAV.ULK1.DN. Top: mCherry fluorescence, bottom: overlay of mCherry fluorescence with brightfield images. Scale bar: 50 µm. **d** Representative immunoblot of cell lysates transduced with given AAV. Expression of ULK1.DN was validated for all samples by blots against its myc-tag. **e**–**j** Top: Representative immunoblots of ULK1, LC3 (high exposure to detect LC3-II), p62, ATG7, and the 56 and 30 kDa bands of ATG5 as well as the corresponding bands of the loading control GAPDH are displayed. Bottom: Quantifications of band intensities of ULK1, LC3-II, p62, ATG7 (all *n* = 6 independent cultures), and the 56 and 30 kDa bands of ATG5 (*n* = 4–5 independent cultures) normalized to GAPDH as loading control. CTR control, RAP treated with rapamycin. Data are presented as single data points and means ± SEM. *P < 0.05, **P < 0.01, ***P < 0.001, N.S. no significant difference, according to one-way analysis of variance (ANOVA) and Sidak’s multiple comparisons test.

To inhibit ULK1 activity, we generated an AAV vector expressing mCherry and ULK1.DN [15], both under the control of human synapsin promotors (AAV.ULK1.DN). As control, we generated an AAV vector, which expresses an untranslated 9(5) fragment [30] instead of ULK1.DN (AAV.CTRL). Both vectors were used to transduce rat cortical neurons (Fig. 1a–c). Overexpression of ULK1.DN was confirmed by immunoblot against its myc-tag (Fig. 1d). To assess potential AAV vector toxicity, we applied different virus titers and quantified the cytotoxicity-related release of adenylate kinase using a toxicity assay. No significant differences in toxicity were detectable between AAV.ULK1.DN and control (Supplementary Fig. 1A, B).

We assessed the expression levels of different autophagic proteins after transduction with AAV.ULK1.DN in basal conditions and after autophagy induction using rapamycin [31]. AAV.ULK1.DN significantly reduced endogenous ULK1 by ~50% compared with control, both with and without addition of rapamycin (Fig. 1e). ULK2 levels remained unchanged (Supplementary Fig. 2). We then quantified the levels of LC3-II, which is bound to the outer membrane of autophagosomes and thus used as a direct marker for autophagic activity [31]. While LC3-II levels significantly increased after addition of rapamycin, transduction with AAV.ULK1.DN attenuated this increase compared with control, indicating an inhibitory effect on autophagy (Fig. 1f). To corroborate this result, we analyzed the expression of p62, which links polyubiquitinated proteins to the autophagic machinery and is therefore used as an indirect marker for autophagy activity [31]. After treatment with rapamycin, we observed a decrease in p62 levels for both AAV.CTRL and AAV.ULK1.DN. Compared with control, AAV.ULK1.DN significantly increased p62 levels for conditions with and without addition of rapamycin (Fig. 1g), confirming an inhibitory effect on autophagy.

In our previous study [12], we found the essential autophagy mediators ATG7 and ATG5 to accumulate in degenerating axons after SCI. No significant differences in ATG7, free (ATG5-30) or ATG12-conjugated ATG5 (ATG5-56) levels, however, were detectable after rapamycin treatment or transduction with AAV.ULK1.DN (Fig. 1h–j).

In summary, we observed a significant regulation of the autophagic proteins ULK1, p62, and LC3-II by AAV.ULK1.DN under autophagy induction with rapamycin, demonstrating an inhibitory effect of AAV.ULK1.DN on autophagy.

### AAV.ULK1.DN does not affect cell survival in vitro

To assess whether the observed modulation of autophagy had an influence on cell survival, we analyzed the expression of the apoptosis marker [32] caspase 3, its cleaved form, and their ratio. No significant differences were detectable after transduction with AAV.ULK1.DN compared with control (Supplementary Fig. 1C–F). In addition, we studied the manifestation of cell death after addition of the apoptosis inductor staurosporine [33]. Transduction with both AAV.ULK1.DN and control resulted in a linear trend to elevated toxicity with increasing staurosporine dosages (Supplementary Fig. 1G), indicating no influence on cell survival by AAV.ULK1.DN.

### AAV.ULK1.DN decreases the number of degeneration bulbs after axotomy in vitro

**Fig. 2:**
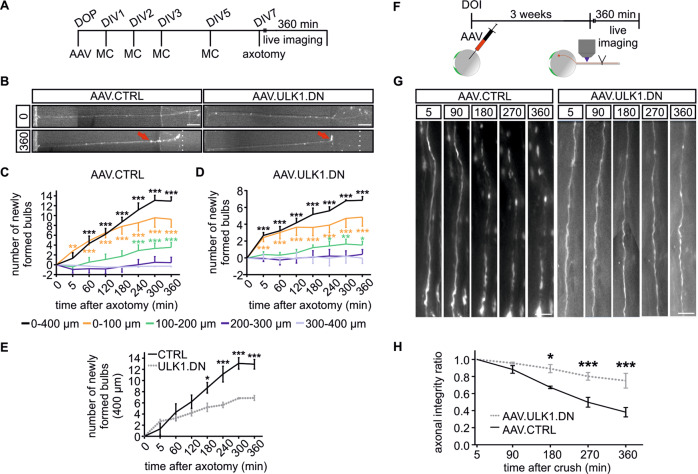
AAV.ULK1.DN reduces the number of axonal degeneration bulbs after axotomy in vitro and attenuates acute axonal degeneration after optic nerve crush in vivo. **a** Scheme of experimental setup for axonal degeneration assays in microfluidic chambers. DOP day of preparation of embryonic day 18 rat cortical neurons, DIV day in vitro, AAV transduction with adeno-associated viral vectors, MC medium change. **b** Representative images of axons growing in microfluidic chambers transduced with viral vectors. Exemplary photomicrographs were taken directly before and 360 min after axotomy. Red arrow: example of axonal bulb used for quantification. Dotted line: area of lesion. Scale bar: 50 µm. **c**, **d** Detailed quantification of the number of newly formed bulbs within different distances (indicated by colors) proximal to the area of lesion at the indicated time points after axotomy and transduction only with AAV.CTRL (**c**, *n* = 4 independent cultures) or AAV.ULK1.DN (**d**, *n* = 4 independent cultures). **e** Quantification of the number of newly formed bulbs within 400 µm distance proximal to the area of lesion at the indicated time points after axotomy and transduction with AAV.CTRL and AAV.ULK1.DN (*n* = 4 independent cultures). **f** Scheme of experimental setup for optic nerve crush (ONC) model. DOI day of intravitreal injection of AAV. **g** Representative images of axons in the optic nerve expressing mCherry after transduction with viral vectors, taken proximal to lesion 5–360 min after axotomy. Scale bar: 20 µm. **h** Quantification of the axonal integrity ratio (ratio of the sum of the length of remaining axonal fragments to the initial axon length) at the indicated time points after ONC and transduction with AAV.CTRL and AAV.ULK1.DN (*n* = 3–4 animals for each viral vector). Data are presented as means ± SEM. *P < 0.05, **P < 0.01, ***P < 0.001, according to two-way repeated measurements (RM) ANOVA and Dunnett’s (**c**, **d**) or Sidak’s multiple comparison’s test (**e**, **h**).

In our previous study, we proposed that ULK1-mediated autophagy induction might represent an important executing mechanism in axonal degeneration [12]. We therefore investigated whether AAV.ULK1.DN might have a beneficial effect on the course of AAD. We used microfluidic culture platforms [25] with transduced primary rat cortical neurons to perform selective axonal lesions in vitro and study AAD via live imaging over 6 h after axotomy (Fig. 2a). As previously published [10], we observed the formation of axonal bulbs, an early sign of axonal degeneration, in a time-dependent manner (Fig. 2b). Quantification of the number of newly formed axonal bulbs within 400 µm proximal to the lesion showed a significant time-dependent increase after transduction with both viral vectors, especially at 0–100 µm proximal to the lesion site (Fig. 2c, d). AAV.ULK1.DN significantly reduced the number of newly formed bulbs starting 3 h after axotomy (Fig. 2e), indicating the attenuation of AAD by ULK1.DN in vitro.

### AAV.ULK1.DN attenuates AAD after ONC in vivo

To confirm the beneficial effect of AAV.ULK1.DN on AAD, we additionally performed rat ONC experiments, which allow to study AAD in vivo [29]. After intravitreal injection of AAV.ULK1.DN and AAV.CTRL to transduce retinal ganglion cells, we performed live imaging over 6 h after ONC (Fig. 2f). As previously published [8], we observed the fragmentation of optic nerve axons and the formation of axonal bulbs in a time-dependent manner (Fig. 2g). We quantified the AIR as the sum length of the remaining axonal fragments divided by the initial axon length (Fig. 2h). Starting 3 h post crush injury, we detected significantly higher AIR values after transduction with AAV.ULK1.DN compared with control, confirming the attenuation of AAD by ULK1.DN in vivo.

### AAD in the optic nerve is attenuated by the ULK1 inhibitor SBI-0206965

**Fig. 3:**
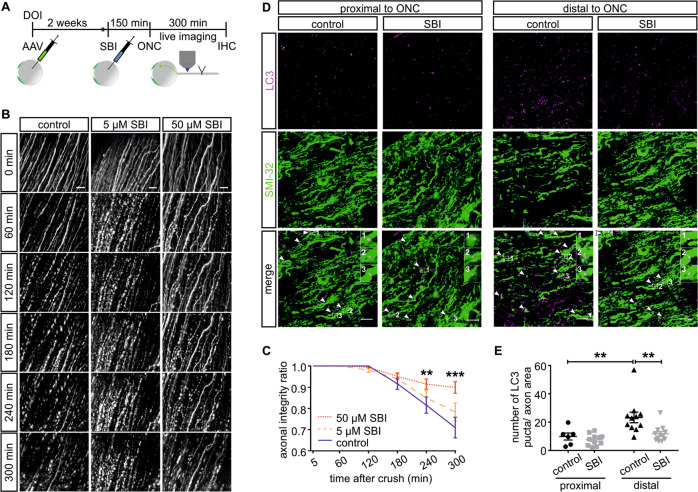
The ULK1 inhibitor SBI-0206965 attenuates acute axonal degeneration and reduces the number of LC3 puncta after optic nerve crush in vivo. **a** Scheme of experimental setup. DOI day of intravitreal injection of AAV.EGFP, SBI intravitreal injection of SBI-0206965 (5 or 50 µM) or control, ONC optic nerve crush, IHC fixation for immunohistochemistry. **b** Representative images of EGFP-labeled optic nerve axons after injection of SBI or control taken proximal to lesion before and 60–300 min after ONC. Scale bar: 30 µm. **c** Quantification of the axonal integrity ratio at the indicated time points after ONC and treatment with SBI or control (*n* = 9–11 animals for each condition). **d** Representative photomicrographs of immunohistochemical staining against the axonal marker SMI32 (green) and the autophagosome marker LC3 (magenta) in the optic nerve after ONC and intravitreal injection of SBI or control. Arrowheads indicate intra-axonal LC3 puncta, insets show examples at higher magnification. Scale bar: 10 µm. **e** Quantification of the number of intra-axonal LC3 puncta normalized to axon area (11–13 visual fields at ~400 µm proximal and distal to lesion, *n* = 3 animals per group). Bars represent single data points and means ± SEM. **P < 0.01, ***P < 0.001, according to two-way RM ANOVA and Tukey’s multiple comparisons test of 50 µM SBI compared with control (**c**) or one-way ANOVA and Tukey’s multiple comparisons test (**e**).

In a translational approach, we additionally tested whether application of the ULK1 inhibitor SBI-0206965 (SBI) [16] led to similar effects on AAD in vivo. Axons of the optic nerve were fluorescently labeled by intravitreal injection of AAV.EGFP. SBI (5 or 50 µM) or control were injected intravitreally 2.5 h before ONC (Fig. 3a). As before, we observed axonal fragmentation in a time-dependent manner after ONC (Fig. 3b). Quantification of the AIR revealed significantly higher values after injection of 50 µM SBI compared with control starting 4 h after crush (Fig. 3c). The lower dosage of 5 µM SBI did not result in a significant difference to control. In summary, we therefore confirmed a protective effect on AAD also by SBI-mediated ULK1 inhibition.

### SBI-0206965 decreases LC3- and increases p62-puncta in the optic nerve

We next aimed to verify that administration of SBI had a similar inhibitory effect on autophagy as AAV.ULK1.DN. Five hours after ONC, we observed a significantly higher number of LC3 puncta distal than proximal to crush in control animals (Fig. 3d, e). SBI treatment significantly lowered the number of distal LC3 puncta, indicating the inhibition of autophagosome formation by SBI. Correspondingly, SBI treatment increased the number of distal intra-axonal p62-puncta (Supplementary Fig. 3), corroborating inhibited autophagosome formation by SBI. Taken together, our data therefore demonstrate that both AAV.ULK1.DN and SBI inhibit autophagy and attenuate AAD after ONC.

### AAV.ULK1.DN attenuates axonal degeneration in a model of SCI in vivo

**Fig. 4:**
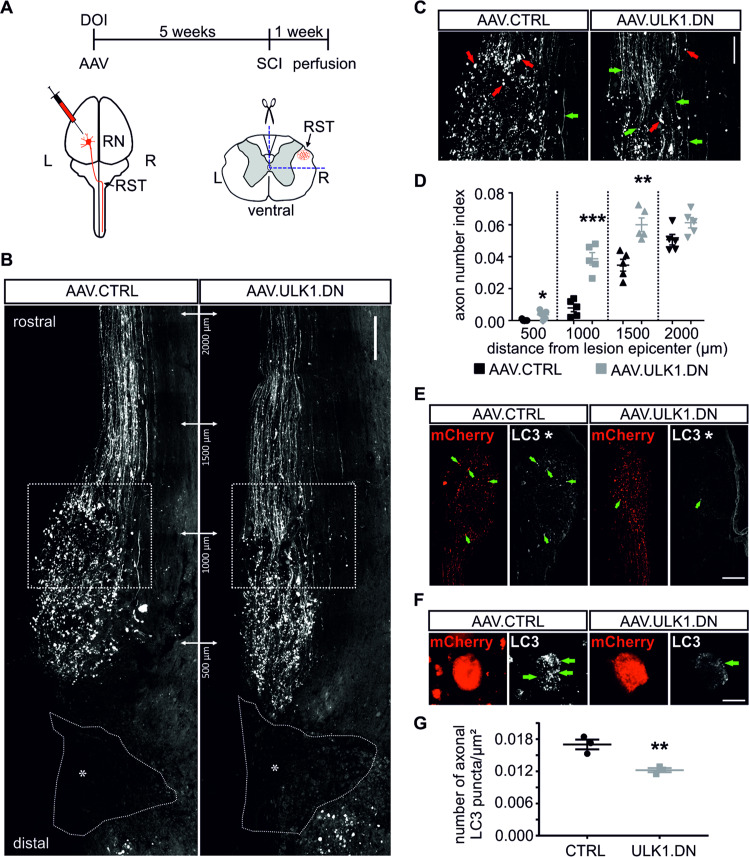
AAV.ULK1.DN attenuates axonal degeneration and decreases the number of axonal LC3 puncta in a model of spinal cord injury in vivo. **a** Scheme of experimental setup for spinal cord injury (SCI) model. DOI day of injection, RN red nucleus, RST rubrospinal tract, L left, R right. **b** Exemplary horizontal sections of thoracic spinal cord transduced with AAV.CTRL and AAV.ULK1.DN after SCI depict areas of lesion and RST up to 2000 µm rostral to lesion. White dashed lines and asterisks indicate lesion areas. White dashed boxes indicate areas shown at higher magnification in **c**. Scale bar: 200 µm. **c** Higher magnification of areas indicated in **b**. Red arrows show formation of bulbs in fragmented axons after lesion. Green arrows indicate intact axons after lesion. Scale bar: 100 µm. **d** Quantification of axon number index (a ratio of nonfragmented (intact) axon numbers to the total number of transduced axons) at the indicated distances from lesion after transduction with AAV.CTRL and AAV.ULK1.DN (*n* = 4–5 animals for each viral vector). **e, f** Exemplary horizontal sections of thoracic spinal cord showing the RST transduced with AAV.CTRL and AAV.ULK1.DN rostral to lesion. mCherry fluorescence is shown in red, immunohistochemical staining against LC3 appears in white. **e** White asterisks indicate lesion. Green arrows indicate axonal bulbs stained for LC3. Scale bar: 400 µm. **f** Higher magnification of axonal bulbs at a distance of 500–1000 µm rostral to the lesion. Green arrows indicate LC3-positive puncta in axonal bulbs. Scale bar: 10 µm. **g** Quantification of the number of axonal LC3 puncta/µm² at a distance of 500–1000 µm rostral to the lesion after transduction with AAV.CTRL and AAV.ULK1.DN (*n* = 3 animals for each viral vector). Data are presented as single data points and means ± SEM. *P < 0.05, **P < 0.01, ***P < 0.001, according to two-tailed unpaired *t*-test.

Having demonstrated attenuated AAD by AAV.ULK1.DN within 6 h after axonal injury, we additionally investigated an effect on axonal degeneration 1 week after axonal lesion. Using our previously reported paradigm [12], we transduced RN neurons in rats and performed a quadrisection of the contralateral spinal cord resulting in the complete transection of the injected RST. One week after lesion, the animals were sacrificed (Fig. 4a). Analysis of their spinal cords showed similar transduction rates for both viral vectors and no differences in the lesion sizes (Supplementary Fig. 4A–E). Similar to our previous study [12], we observed the fragmentation of axons and formation of axonal bulbs (Fig. 4b, c). We quantified the axon number index (a ratio of nonfragmented (intact) axon numbers to the total number of transduced axons) and found significantly higher values at 500, 1000, and 1500 µm rostral to the lesion after transduction with AAV.ULK1.DN compared with control (Fig. 4d), demonstrating the attenuation of axonal degeneration by ULK1.DN. The evaluation of Wallerian degeneration, however, showed a nearly complete axonal fragmentation distal to the lesion and no difference between both vectors (Supplementary Fig. 4F, G). Taken together, AAV.ULK1.DN therefore also protects from chronic axonal degeneration after axonal lesion.

### AAV.ULK1.DN decreases axonal LC3 puncta after SCI in vivo

To confirm an inhibitory effect of AAV.ULK1.DN on autophagy also after SCI, we analyzed the number of axonal LC3 puncta (Fig. 4e, f). We observed a significantly lower number of axonal LC3 puncta rostral to the lesion in AAV.ULK1.DN-injected rats compared with control animals (Fig. 4g). AAV.ULK1.DN therefore leads to a significant reduction of autophagosomes also after SCI.

### Proteomic analysis reveals that AAV.ULK1.DN regulates proteins involved in translation and splicing

**Fig. 5:**
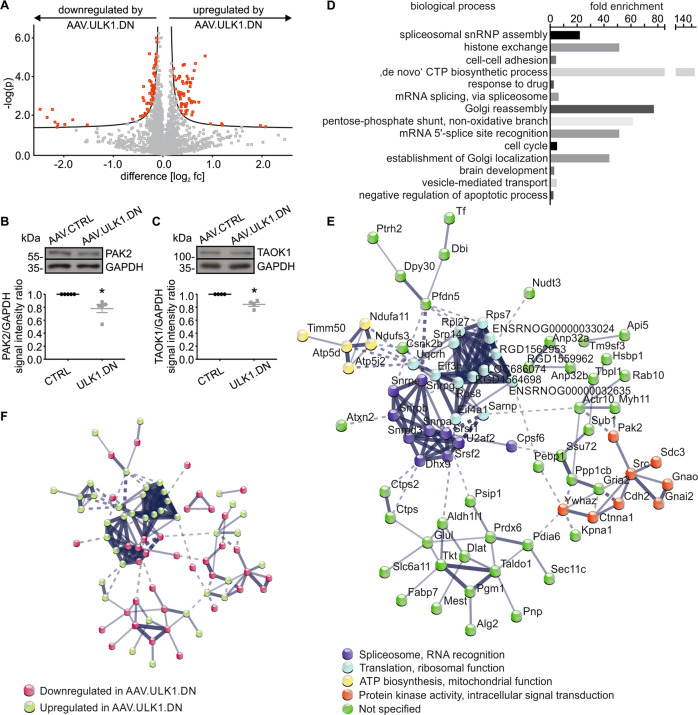
Quantitative proteomic profiling reveals AAV.ULK1.DN-mediated regulation of biological processes and protein interactions. Lysates obtained from E18 rat cortical neurons on DIV 8 after transduction with AAV.CTRL or AAV.ULK1.DN were subjected to proteomics analysis. **a** Volcano plot showing all 1988 quantifiable proteins in SWATH-MS, 122 significantly regulated proteins according to two-sided *t*-test with permutation-based FDR assessment (FDR 0.1, s0 = 0.05) appear in red (*n* = 2 independent cultures, three technical replicates for each condition for each experiment). **b**, **c** Immunoblotting against two selected proteins regulated in quantitative proteomic profiling was performed to validate the results. Representative western blots of PAK2 and TAOK1 are shown at the top, the quantifications of band intensities normalized to GAPDH as loading control are depicted at the bottom (*n* = 4–5 independent cultures different to those used for the proteomics analysis). Data are presented as single data points and means ± SEM. *P < 0.05 according to one-sample *t*-test. **d** Biological processes annotated to significantly regulated proteins by AAV.ULK1.DN according to enrichment analysis of functional annotations in Gene Ontology. The fold enrichment value is given for each biological process. Only significantly regulated biological processes are shown. **e** Protein network map of all significantly regulated proteins after transduction with AAV.ULK1.DN in comparison with AAV.CTRL showing a significant number of interactions (STRING database enrichment *P* value = 6.93E−07). Four clusters according to k-Means clustering are highlighted by different colors. **f** The same protein network map as in **e** highlighting down- and upregulated proteins.

AAV.ULK1.DN inhibits autophagy both in vitro and in vivo, and we have previously demonstrated that autophagy inhibition protects from lesion-induced axonal degeneration [8, 11]. Yet, in addition to its canonical function in autophagy, ULK1 interacts with a vast array of proteins [34]. We thus investigated whether the regulation of further molecular mechanisms, in addition to autophagy, might be responsible for the AAV.ULK1.DN-mediated attenuation of axonal degeneration demonstrated in this study. We performed a quantitative mass spectrometry-based proteome analysis of rat cortical neurons transduced with AAV.ULK1.DN or AAV.CTRL. Out of 1988 quantifiable proteins, 122 proteins were significantly regulated by AAV.ULK1.DN (Fig. 5a). To validate the results of the proteomic approach, immunoblot analyses of PAK2 and TAOK1 were performed, which were among the ten proteins with strongest up- or downregulation (Supplementary Fig. 5) and are implicated in processes associated with the pathophysiology of axonal degeneration [35, 36]. Significantly lower levels of PAK2 and TAOK1 were detected after transduction with AAV.ULK1.DN (Fig. 5b, c).

To investigate the functions of all significantly regulated proteins, we performed an enrichment analysis of GO annotations to biological processes and cellular components. Interestingly, we observed a significant regulation of processes associated with the spliceosome as well as the Golgi system (Fig. 5d, Supplementary Tables 1 and 2). In a STRING database analysis, we detected a significant number of interactions amongst the regulated proteins revealing four clusters, grouped by roles in translational processes, ATP biosynthesis/mitochondrial function, the spliceosome, and protein kinase activity (Fig. 5e). Proteins involved in translational processes and ATP biosynthesis/mitochondrial function were mainly upregulated after transduction with AAV.ULK1.DN, whereas about one half of the proteins associated with spliceosome and protein kinase activity were upregulated and the other half showed downregulation (Fig. 5f). In summary, the proteomics analysis outlined a prominent regulation of proteins that are involved in translation and splicing by AAV.ULK1.DN.

### AAV.ULK1.DN increases translation through enhanced mTOR signaling

**Fig. 6:**
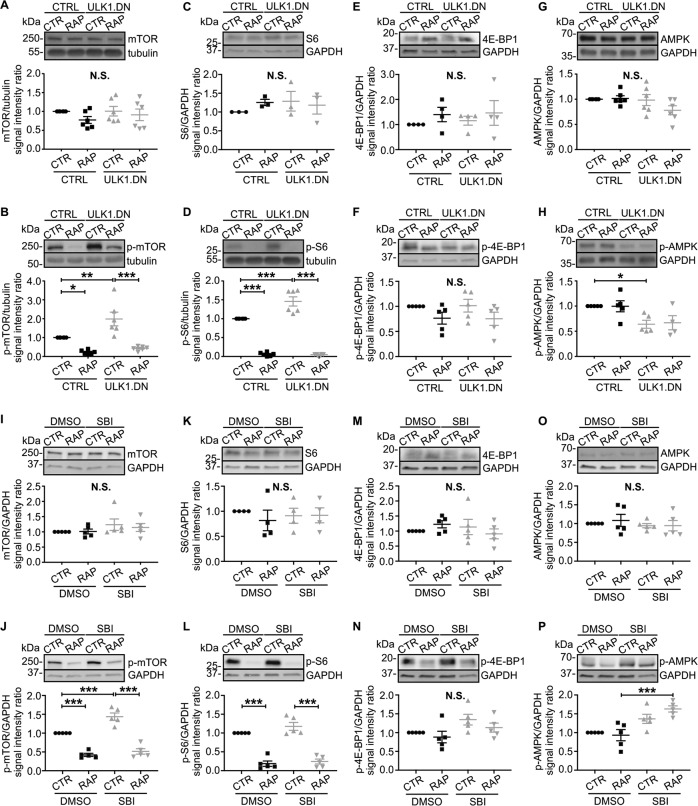
Both AAV.ULK1.DN and the ULK1 inhibitor SBI-0206965 enhance mTOR activation. Lysates were obtained from E18 rat cortical neurons on DIV 8 after transduction with AAV.ULK1.DN or AAV.CTRL. Alternatively, cells were treated with the ULK1 inhibitor SBI-0206965 (SBI, 5 μM) or DMSO as control for 30 min before lysis. RAP addition of rapamycin (750 nM) 24 h before lysis. **a**–**h** Top: Representative immunoblots of mTOR, p-mTOR, S6, p-S6, 4E-BP1, p-4E-BP1, AMPK, p-AMPK, and the corresponding bands of the loading controls tubulin or GAPDH after transduction with AAV.ULK1.DN or AAV.CTRL are shown. Bottom: Quantifications of the band intensities of mTOR, p-mTOR, p-S6, AMPK (all *n* = 6 independent cultures), p-4E-BP1 (*n* = 5 independent cultures), p-AMPK (*n* = 4–5 independent cultures), 4E-BP1 (*n* = 4 independent cultures), and S6 (*n* = 3 independent cultures) normalized to tubulin or GAPDH as loading controls. CTR control, RAP treated with rapamycin. **i–p** Top: Representative immunoblots of mTOR, p-mTOR, S6, p-S6, 4E-BP1, p-4E-BP1, AMPK, p-AMPK, and the corresponding bands of the loading control GAPDH after treatment with SBI or DMSO as control are shown. Bottom: Quantifications of the band intensities of mTOR, p-mTOR, p-S6, AMPK, p-AMPK, 4E-BP1, p-4E-BP1 (all *n* = 5 independent cultures), and S6 (*n* = 4 independent cultures) normalized to GAPDH as loading control. CTR control, RAP treated with rapamycin. Data are presented as single data points and means ± SEM. *P < 0.05, **P < 0.01, ***P < 0.001, N.S. no significant difference, according to one-way ANOVA and Sidak’s multiple comparisons test.

Next, we aimed to investigate the distinct upregulation of translation-associated proteins by AAV.ULK1.DN more closely, as dysregulated protein translation has been implicated in axonal degenerative cascades [37–40]. Furthermore, a close interaction between ULK1 and the translational master regulator mTOR is well characterized [41]. We thus analyzed the expression level of mTOR after transduction with AAV.ULK1.DN and AAV.CTRL. Equal values were detectable in both basal conditions and after administration of rapamycin (Fig. 6a). As mTOR activity is finely regulated by phosphorylation [41], we additionally studied the levels of phosphorylated mTOR (p-mTOR, Ser2448), which corresponds to activated mTOR [42]. We found significantly higher levels of p-mTOR after transduction with AAV.ULK1.DN (Fig. 6b), indicating increased mTOR activation. Administration of rapamycin markedly reduced p-mTOR levels after transduction with both vectors. To confirm higher mTOR activation, we analyzed the levels of S6 ribosomal protein (S6) and its phosphorylated form (p-S6), which is a downstream target of active mTOR [43]. Phosphorylation of S6 induces increased translation of mRNA transcripts that include ribosomal proteins and elongation factors required for translation [43–45]. Transduction with AAV.ULK1.DN did not alter total S6 expression but significantly increased p-S6 levels, confirming enhanced mTOR-dependent translation (Fig. 6c, d). The expression of the translational repressor [46] 4E-BP1 and its phosphorylated form (Thr37/46) were not significantly altered (Fig. 6e, f), indicating that ULK1.DN acts on translation independent of 4E-BP1.

To better understand the connection between ULK1.DN and mTOR, we additionally analyzed the expression of AMPK, which interacts with ULK1 and inhibits mTOR and mTOR-dependent translation [41]. Quantification of the main subunit AMPKα showed equal values in all conditions (Fig. 6g). However, analysis of the phosphorylated (Thr172), thus active form of AMPKα [47, 48] revealed significantly lower levels after transduction with AAV.ULK1.DN (Fig. 6h), suggesting that a reduction in AMPK-mediated negative feedback might contribute to the positive effects of ULK1 inhibition on mTOR signaling.

### SBI-0206965-mediated ULK1 inhibition leads to mTOR activation

To validate our findings after transduction with AAV.ULK1.DN, we performed acute (30 min) pharmacological ULK1 inhibition using SBI in cortical neurons and studied the effects on the mTOR pathway. Consistently, treatment with SBI did not alter total mTOR expression but significantly increased the levels of p-mTOR compared with control (Fig. 6i, j). The total levels of S6 and 4E-BP1 were not significantly regulated (Fig. 6k, m), while the analysis of p-S6 and p-4E-BP1 showed nonsignificant trends to higher levels after SBI administration (Fig. 6l, n). The expression of AMPKα remained unchanged (Fig. 6o). Surprisingly, SBI led to increased p-AMPK levels in rapamycin-treated conditions (Fig. 6p), arguing against a crucial role of reduced AMPK activity for increased mTOR activity through ULK1 inhibition, at least in this acute paradigm. Taken together, these data suggest that increased translation following ULK1 inhibition is mediated through an mTOR-dependent mechanism, representing an additional molecular mediator of its degeneration-attenuating effect.

### AAV.ULK1.DN differentially regulates the splicing of degeneration-associated genes

**Fig. 7:**
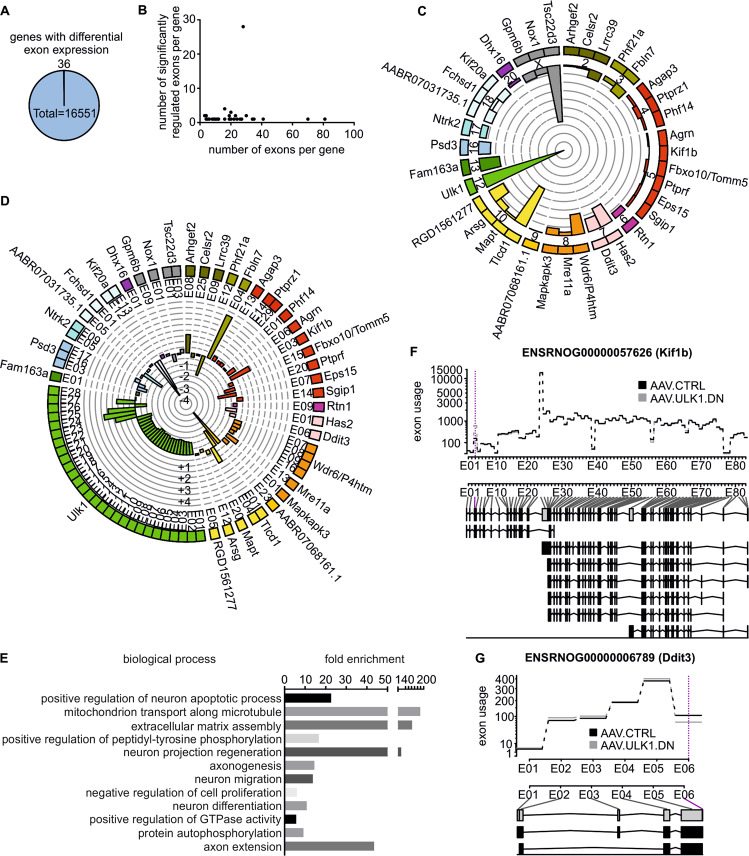
Differential exon expression analysis reveals AAV.ULK1.DN-mediated splicing of genes associated with axonal degeneration. Lysates obtained from E18 rat cortical neurons on DIV 8 after transduction with AAV.CTRL or AAV.ULK1.DN were subjected to differential exon expression analysis. **a** Pie chart showing the number of genes with significantly (FDR-corrected *P* value < 0.05) differential exon expression out of all sequenced genes after transduction with AAV.ULK1.DN (*n* = 3 independent cultures). **b** Graph showing the number of significantly regulated exons by AAV.ULK1.DN compared with the total number of exons per gene. **c** Graph showing the names and number of genes with differential exon expression per chromosome (2-X) after transduction with AAV.ULK1.DN. The percentage of differentially expressed exons out of the total exon number per gene is indicated by bars, circular lines indicate percentages from 0 to 100%. **d** Graph showing differentially expressed exons per gene (e.g., E01: exon 1). Bars indicate the log2 fold change (fc) in expression for each exon by AAV.ULK1.DN. Circular gray lines indicate log2 fc from −4.5 to 4.5. **e** Biological processes annotated to genes with significantly differential exon expression by AAV.ULK1.DN according to enrichment analysis in Gene Ontology. The fold enrichment value is given for each biological process. Only significantly regulated biological processes are shown. **f**, **g** Top: Graphs showing the exon usage of two genes (*Kif1b, Ddit3*) after transduction with AAV.CTRL and AAV.ULK1.DN. Dotted magenta lines indicate significant differences (FDR-corrected *P* value < 0.05). Bottom: Overview of different transcripts of the same gene, the first row indicates the full transcript.

In addition to proteins involved in translation, we surprisingly identified a pronounced regulation of splicing-associated proteins in our proteomics analysis. Defects in splicing have been closely linked to neurodegenerative diseases such as ALS [49, 50]. We therefore investigated whether the attenuation of axonal degeneration by AAV.ULK1.DN might additionally be mediated by an effect on splicing. We performed a differential exon expression analysis of rat cortical neurons transduced with AAV.ULK1.DN or AAV.CTRL. Thirty-six genes with significantly differential exon expression were found after transduction with AAV.ULK1.DN (Fig. 7a), particularly on chromosomes 5 and 10 (Fig. 7c). Expectedly, the strongest change in the relative number of regulated exons per gene was detected for *Ulk1*. Overall 24/28 *Ulk1* exons were downregulated and 4/28 were upregulated (Fig. 7b–d). In line with this, AAV.ULK1.DN led to a significantly higher gene expression of *Ulk1* (Supplementary Fig. 6), both analyses reflecting the overexpression of ULK1.DN.

Investigation of the molecular function, cellular component, and biological processes of the genes with differential exon expression showed a consistent enrichment of GO terms associated with neurite outgrowth and microtubules (Fig. 7e, Supplementary Tables 3–5). Closer evaluation revealed a significant differential exon usage for the genes *Ddit3* and *Kif1b* by AAV.ULK1.DN (Fig. 7f, g), both of which have previously been implicated in the pathophysiology of axonal degeneration [51, 52]. In summary, we have therefore uncovered differential splicing of specific degeneration-associated genes as an additional mechanism that mediates the degeneration-attenuating effect of ULK1.DN.

## Discussion

ULK1 is critical for the initiation of the autophagy cascade [13]. In this study, we overexpress ULK1.DN, which is comprised of only the C-terminal amino acids 829–1051 and lacks the kinase domain [15]. ULK1.DN is thus unable to phosphorylate its substrates and activate autophagy, resulting in a dominant-negative effect [15]. Here, we additionally demonstrate that ULK1.DN reduces the expression of endogenous ULK1.

ULK1.DN moderately inhibits autophagy activation after administration of rapamycin. This is in line with experiments using ULK1.siRNA and ULK1 knockout, where a moderate inhibitory effect on autophagy and no complete blockage was seen [13, 15, 53]. Under unstimulated conditions, ULK1.DN leads to p62 accumulation but elicits no effect on LC3-II, ATG5, or ATG7 levels. LC3 was found unchanged in previous experiments [13, 15] using ULK1.siRNA and ULK1.DN, but the levels of p62, ATG5, or ATG7 were not addressed in these studies. ULK1-dependent ATG5/ATG7-independent autophagy has been described before in mammalian cells [54, 55]. Our data could thus indicate an effect of ULK1.DN on autophagy that is independent of these autophagy regulators under unstimulated conditions, leading to accumulation of p62, while conventional LC3-mediated autophagy is affected only after rapamycin stimulation.

Given the described interactions between autophagy and apoptosis [56], we evaluated the effects of ULK1.DN in a cell death paradigm. ULK1.DN did not alter cleaved caspase 3 levels nor adenylate kinase release—neither under unstimulated conditions nor after induction of apoptosis with staurosporine. Previously, an ULK1 agonist has been reported to trigger cell death, while ULK1.siRNA rescued this effect [57]. On the other hand, small-molecule inhibition of ULK1 by SBI attenuated autophagy-dependent cell survival [16]. Depending on the context and model, ULK1 can thus exert different effects on cell survival.

Inhibition of ULK1 by ULK1.DN protects cortical neurons in vitro and retinal ganglion cells in vivo from AAD over 6 h after axonal injury, which we confirm by application of the ULK1 inhibitor SBI. We additionally demonstrate a protective effect of ULK1.DN on rubrospinal projections 1 week after spinal cord lesion. Previously, we have described the accumulation of autophagosomes and autophagic proteins including ULK1 in degenerated axons after SCI [12]. Accumulated autophagosomes and higher p-ULK1 levels were also reported in rubrospinal neurons after spinal cord hemisection, while blockage of autophagy using 3-MA reduced neuronal death [58]. However, these results were merely descriptive, leaving the functional role of ULK1 in axonal degeneration unclear. Recently, we have demonstrated ULK1.DN-mediated protection of dopaminergic nigral neurons and nigro-striatal projections against MPTP-induced degeneration in a mouse model of Parkinson’s disease [17]. Here, we significantly advance these findings, providing to our best knowledge the first evidence that inhibition of ULK1 function protects three different types of neurons against axonal degeneration after traumatic axonal lesion, both in an acute and chronic paradigm.

**Fig. 8:**
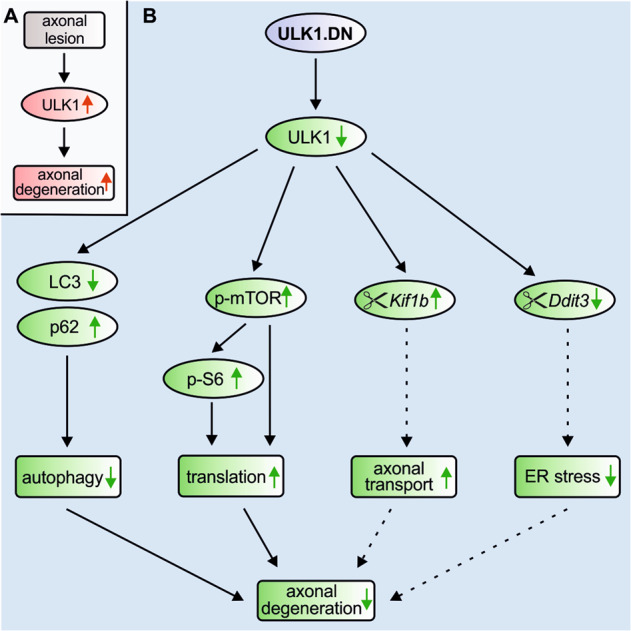
Schematic drawing of the molecular mechanisms by which ULK1.DN attenuates axonal degeneration. **a** We have previously demonstrated the upregulation of ULK1 and autophagy during axonal degeneration after axonal lesion [12]. **b** In this study, ULK1.DN-mediated inhibition of ULK1 function significantly protects axons from degenerating in different models of lesion-induced axonal degeneration. Mechanistically, ULK1.DN decreases autophagy as demonstrated by altered levels of LC3 and p62. Autophagy inhibition attenuated axonal degeneration in our previous studies [8, 11] and is thus one of the mechanisms by which ULK1.DN exerts neuroprotective effects. Furthermore, ULK1.DN enhances translation through increased mTOR and S6 signaling. Increased translation represents a second mediator of the degeneration-attenuating effect of ULK1.DN, as dysregulated translation is implicated in axonal degenerative cascades [37, 38, 40, 59]. In addition, we demonstrate that ULK1.DN modulates the differential splicing of *Kif1b*, which plays a role in axonal transport and has previously been shown to be involved in axonal degeneration [52, 62, 63]. Similarly, *Ddit3* is differentially spliced by ULK1.DN, which mediates ER stress and has been implicated in models of neurodegeneration [51, 66–68]. Differential splicing of these genes is therefore another mechanism by which ULK1.DN mediates the attenuation of axonal degeneration.

Combining the attenuation of autophagy by ULK1.DN demonstrated in this study and our previously published evidence showing the beneficial effects of autophagy inhibition on axonal degeneration [8, 11], it is likely that reduced autophagy is one of the mechanisms by which ULK1.DN counteracts axonal degeneration (Fig. 8). However, in addition to its canonical function in autophagy, ULK1 has been described to interact with a plethora of other proteins [34]. Here, proteomics analysis identifies foremost changes in proteins involved in translation and splicing by ULK1.DN. We further connect the upregulation of translational proteins to increased mTOR signaling. We also observed increased mTOR activity after injection of ULK1.DN into the midbrain [17]. An interaction between ULK1 and the translational master regulator mTOR is well characterized [41] and dysregulated translation has been implicated in axonal degenerative cascades [37, 38, 40, 59]. An additional mechanism by which ULK1.DN protects axons from degeneration is thus increased protein translation (Fig. 8). The exact mechanism of how ULK1.DN-induced translation exerts neuroprotective effects remains to be determined; a potential means might be increased local translation of axon-protective molecules.

Most interestingly, ULK1.DN leads to a pronounced regulation of spliceosome-associated proteins. Defects in splicing have been linked to numerous neurological disorders including neurodegenerative diseases [49], very prominently in the pathology of ALS [60] and spinal muscular atrophy [61]. Transcriptomic analysis reveals differential splicing by ULK1.DN, especially of microtubule-related genes, which are implicated in the pathophysiology of neurodegenerative disorders [62]. To the best of our knowledge, we thus provide here the first description of a role of ULK1 in splicing. Specifically, the axonal transporter *Kif1b* displays significantly differential exon expression. Kif1b has been revealed mutated in the neuropathy Charcot–Marie–Tooth disease type 2A and reduced retinal expression of Kif1b mediated retinal ganglion cell decline in a mouse model of chronic glaucoma [52, 63]. Intriguingly, ULK1 has previously been connected to kinesin 1-dependent axonal transport in drosophila [64] and in vitro assays have shown that alternative splice forms of *Kif1b* induce higher activity and affinity for microtubules [65]. One additional explanation for the observed attenuation of axonal degeneration by ULK1.DN could thus be increased axonal transport through modulated splicing of *Kif1b* (Fig. 8).

Furthermore, we observed differential exon expression of *Ddit3*, which mediated ER stress following SCI, while its deletion improved functional recovery [51, 66]. Ddit3 has also been implicated in retinal ganglion cell death after axonal injury and its inhibition has been protective against glaucomatous neurodegeneration [67, 68]. An additional explanation for the observed attenuation of axonal degeneration by ULK1.DN might thus be reduced ER stress through modulated splicing of *Ddit3* (Fig. 8).

In conclusion, this study demonstrates that ULK1 inhibition markedly attenuates the process of axonal degeneration after lesion in vitro and in vivo. In addition to decreased autophagy, inhibition of ULK1 leads to an mTOR-mediated increase in translation and differential splicing of the degeneration-associated genes *Kif1b* and *Ddit3*. We propose that modulation of splicing, increased translation, and inhibition of autophagy are crucial mechanisms by which ULK1 inhibition protects against axonal degeneration. ULK1 thus represents a novel therapeutic target in traumatic and degenerative diseases of the CNS.

## Supplementary information

Supplemental Material

Supplementary Figure S1

Supplementary Figure S2

Supplementary Figure S3

Supplementary Figure S4

Supplementary Figure S5

Supplementary Figure S6

## Data Availability

The mass spectrometry data have been deposited to the ProteomeXchange Consortium via the PRIDE [69] partner repository with the dataset identifier PXD011862. The RNA sequencing and transcriptomic data are available at GEO accession number GSE123687.
