# Supplementary material for: Inhibition of the autophagic protein ULK1 attenuates axonal degeneration in vitro and in vivo, enhances translation, and modulates splicing
Source: Cell Death Differ. 2020 Apr 27;27(10):2810–27. doi: 10.1038/s41418-020-0543-y (PMC7493890; doi:10.1038/s41418-020-0543-y)
Supplement: Supplementary file 1 — Supplemental Material [file 41418_2020_543_MOESM1_ESM.docx]

# Supplementary Material

**Title: Inhibition of the autophagic protein ULK1 attenuates axonal degeneration *in vitro* and *in vivo,* enhances translation, and modulates splicing**

**Authors:**

Björn Friedhelm Vahsen^a,*^, Vinicius Toledo Ribas^b,*^, Jonas Sundermeyer^a^, Alexander Boecker^c^, Vivian Dambeck^a,d,e^, Christof Lenz^f,g^, Orr Shomroni^h^, Lucas Caldi Gomes^a^, Lars Tatenhorst^a,d,e^, Elisabeth Barski^a^, Anna-Elisa Roser^a,e^, Uwe Michel^a^, Henning Urlaub^f,g^, Gabriela Salinas^h^, Mathias Bähr^a,e^, Jan Christoph Koch^a,e^, Paul Lingor^a,d,e,i^

**Corresponding Author:**

Paul Lingor, Department of Neurology, Rechts der Isar Hospital of the Technical University Munich, Munich, Germany. Phone: +49 89 4140 8257. Fax: +49 89 4140 4867. Email: paul.lingor@tum.de

This Supplementary Material contains Supplementary Materials and Methods, Supplementary Figure Legends, and Supplementary Tables 1-5.

## Supplementary Materials and Methods

## Neuronal cell culture, viral transduction and toxicity assay

Primary rat cortical neurons were prepared from embryonic day 18 (E18) rats as described before^1^. In brief, pregnant Wistar rats were sacrificed by CO_2_ intoxication. Dissected embryonic cortices were trypsinized (Sigma-Aldrich, St. Louis, MO, USA) for 12 min at 37°C, and then triturated with a fire-polished Pasteur pipette. 4x10^5^ cortical neurons were seeded in 24-well-plates after poly-L-ornithine and laminin (both Sigma-Aldrich) coating and cultured in cortex medium at 37°C and 5% CO_2_. On day *in vitro* (DIV) 1, cells were transduced with AAV.CTRL (5x10^6^ transducing units (TU)) and AAV.ULK1.DN (9x10^6^ TU), resulting in equal transduction rates (70-80%) and only minor toxicity. Medium changes were performed every other day.

To induce autophagy, rapamycin (750 nM, Sigma-Aldrich) was added to the medium in selected conditions on DIV 7 for 24 hours. To evaluate a possible protective effect against cell death, staurosporine (Sigma-Aldrich) was added to the medium at different concentrations (30 nM, 100 nM, 300 nM) on DIV 7 for 24 hours. 30 min before lysis on DIV 8, cells were treated with SBI-0206965 (5 µM, dissolved in DMSO, Sigma-Aldrich) or with DMSO as vehicle control in selected conditions.

## Cell lysis and Western blot analysis

Cortical neurons were lysed in ice-cold lysis buffer on DIV 8. Protein lysates were homogenized using ultrasound sonication. After centrifugation at 4°C and 14.0 rpm for 30 min, the protein content of each sample was determined. Equal amounts of protein (10-30 µg) were loaded onto each lane and separated by sodium dodecyl sulfate polyacrylamide gel electrophoresis (SDS-PAGE). Hereafter, proteins were transferred to a nitrocellulose (AppliChem, Darmstadt, Germany) or polyvinylidene difluoride membrane (GE Healthcare Life Sciences, Boston, MA, USA) at 4°C and 100 V for 2 h or at 20 V overnight (ON). After blocking with 5% milk or 5% bovine serum albumin (BSA) in Tris-buffered saline/0.1% Tween-20 (TBS-T, all AppliChem) for 1 h at room temperature (RT), membranes were incubated with primary antibodies diluted in 5% milk or 5% BSA in TBS-T at 4°C ON. This was followed by incubation with corresponding horseradish peroxidase (HRP)-coupled or fluorescent secondary antibodies at RT for 1 h. After application of enhanced chemiluminescence solution (250 mM luminol (Merck, Darmstadt, Germany), 90 mM p-coumaric acid, 1M Tris pH 8.5 (both AppliChem), 30% hydrogen peroxide (Sigma-Aldrich)), signal detection was performed within the linear range on X-Ray films (GE Healthcare Life Sciences) developed in a Curix 60 Developer (Agfa, Mortsel, Belgium) or using the Fusion Pulse 6 imager (Vilber Lourmat, Collegien, France) equipped with Evolution-Capt software. Fluorescent antibodies were visualized on the Odyssey Sa Imaging System (LI-COR Biosciences, Lincoln, NE, USA) equipped with Image studio software. Band intensities were quantified using ImageJ software (open freeware provided by the NIH, Bethesda, MD; https://imagej.nih.gov/ij/). Target protein band intensities were normalized to the band intensities of the housekeeping genes tubulin or GAPDH.

## RNA isolation

For transcriptomic analysis, cortical neurons were lysed with TRI-reagent (Sigma-Aldrich) for total RNA extraction on DIV 8. Organic/aqueous phase-separation was achieved using 1-bromo-3-chlor-propane (Sigma-Aldrich) and centrifugation at 12000 x g at 4°C for 15 min. Subsequently, RNA precipitation was performed by adding 250 µl isopropanol (AppliChem) and 1 µl Glycoblue Coprecipitant 15 mg/ml (ThermoFisher Scientific) to the aqueous phase in a new Eppendorf tube. After incubation ON and centrifugation at 12000 x g and 4°C for 30 min, the RNA pellet was washed twice with 75% ice-cold ethanol, reconstituted with 20 µl DEPC-treated water (Sigma-Aldrich) and kept at -80°C until further use.

## Differential exon and gene expression analyses

For raw read and quality check, sequence images were transformed with Illumina software BaseCaller to BCL files, which was demultiplexed to fastq files with bcl2fastq v2.17.1.14. The sequencing quality was asserted using FastQC^2^ version 0.11.5. For mapping and normalization, sequences were aligned to the reference genome Rattus Norvegicus (rn6 version 93, <https://www.ensembl.org/Rattus_norvegicus/Info/Index>) as paired-end reads using the STAR aligner^3^ allowing for 2 mismatches within 50 bases.

For differential exon usage analysis, reads were counted for individual exons and analyzed in the R/Bioconductor environment (version 3.4.2, www.bioconductor.org) using the DEXSeq package^4^ version 1.22.0. The exon usage was calculated as the expression of each exon with respect to the expression of other exons in the same gene, i.e. ${eu}_{i\in I}=\frac{{ee}_{i\in I}}{\sum_{j\in I, j\neq i} {ee}_{j}}$, where ${eu}_{i\in I}$ is the exon usage of exon i in gene I, ${ee}_{i\in I}$ is the exon expression of exon i in gene I and $\sum_{j\in I, j\neq i} {ee}_{j}$ is the sum of expressions of exons j in gene I which are no exon i. Candidate genes were filtered using a False Discovery Rate (FDR)-corrected p-value<0.05. Gene annotation was performed using Rattus Norvegicus entries via biomaRt R package^5^ version 2.32.1.

For differential expression analysis, read counting was performed using featureCounts^6^ (version 1.4.5-p1) using parameters “-p -P” to count the number of fragments instead of individual reads. Read counts were then analyzed in the R/Bioconductor environment using the DESeq2 package^7^ version 1.14.1. Candidate genes were filtered using an absolute log2 fold-change>1 and FDR-corrected p-value<0.05. Gene annotation was performed using Rattus Norvegicus entries via biomaRt.

## Proteomics analysis

Cells were lysed, and the protein content of each sample was determined as described above. 100 µg protein from each sample prepared for quantitative proteomic profiling were precipitated with acetone (AppliChem) at -20°C ON. After centrifugation, dry pellets were stored at -20°C until further analysis. Protein lysates were separated using gel electrophoresis, digested with trypsin and extracted from the gel.

Protein digests were analyzed on a nanoﬂow chromatography system (Eksigent nanoLC425, Dublin, CA, USA), hyphenated to a hybrid triple quadrupole-TOF mass spectrometer (TripleTOF 5600+) equipped with a Nanospray III ion source (Ionspray Voltage 2400 V, Interface Heater Temperature 150°C, Sheath Gas Setting 12) and controlled by Analyst TF 1.7.1 software build 1163 (all AB Sciex, Framingham, MA, USA). In brief, peptides were dissolved in loading buffer (2% acetonitrile, 0.1% formic acid in water) to a concentration of 0.3 µg/µl. For each analysis, 1.5 g protein were enriched on a self-packed precolumn (0.15 mm ID x 20 mm, Reprosil-Pur120 C18-AQ 5 µm, Dr. Maisch, Ammerbuch-Entringen, Germany) and separated on an analytical RP-C18 column (0.075 mm ID x 200 mm, Reprosil-Pur 120 C18-AQ, 3 µm, Dr. Maisch) using a 100 min linear gradient of 5-35% acetonitrile/0.1% formic acid (v:v) at 300 nl min-1.

Qualitative LC/MS/MS analysis was performed using a Top30 data-dependent acquisition method with an MS survey scan of m/z 380–1250 accumulated for 250 ms at a resolution of 35 000 full width at half maximum (FWHM). MS/MS scans of m/z 180–1500 were accumulated for 100 ms at a resolution of 17,500 FWHM and a precursor isolation width of 0.7 FWHM, resulting in a total cycle time of 3.4 s. Precursors above a threshold MS intensity of 200 cps with charge states 2+, 3+, and 4+ were selected for MS/MS, the dynamic exclusion time was set to 15 s. MS/MS activation was achieved by CID using nitrogen as a collision gas and the manufacturer’s default rolling collision energy settings. Two biological replicates per sample were analyzed to construct a spectral library.

For quantitative SWATH analysis, MS/MS data were acquired using 100 variable size windows^8^ across the 400-1200 m/z range. Fragments were produced using rolling collision energy settings for charge state 2+, and fragments acquired over an m/z range of 180–1500 for 40 ms per segment. Including a 250 ms survey scan this resulted in an overall cycle time of 4.3 s. Three replicate injections were acquired for each of the two biological replicates of the four samples.

Protein identiﬁcation was achieved using ProteinPilot Software version 5.0 build 4304 (AB Sciex) at “thorough” settings. A total of 436,865 MS/MS spectra from the combined qualitative analyses were searched against the UniProtKB Rattus norvegicus reference proteome (revision 04-2016, 31,606 entries) augmented with a set of 51 known common laboratory contaminants to identify 2,440 proteins at an FDR of 1%.

SWATH peak extraction was achieved in PeakView Software version 2.1 build 11041 (AB Sciex) using the SWATH quantitation microApp version 2.0 build 2003. Following retention time correction on endogenous peptides spanning the entire retention time range, peak areas were extracted using information from the MS/MS library, resulting in 505,568 features extracted at an FDR of 1%^9^. The resulting peak areas were then summed to peptide and ﬁnally protein area values, which were used for further statistical analysis. Statistical evaluation of protein quantitation data was performed using Perseus 1.5.0.15 software (MPI for Biochemistry, Martinsried, Germany). Following sample grouping and logarithmic transformation, 122/1988 proteins showed significant fold changes as assessed by a two-sided T-test with permutation-based FDR assessment (FDR 0.1, s0=0.05).

## Microfluidic chambers and axotomy *in vitro*

The microfluidic chamber systems consist of four wells, two of which are connected by main channels. The first main channel is used as ‘soma compartment’, the second one as ‘axonal compartment’. Small microgrooves (7 µm wide, 3 µm high, 450 µm long) connect these two main channels. The chambers were produced based on previously published protocols^1,10,11^. Briefly described, poly(dimethylsiloxane) prepolymer and cross-linker (Sylgard 184 silicone elastomer kit, Dow Corning, Midland, MI, USA) were mixed 10:1, poured onto a master mold (fabricated by photolithograpy) and cured at 60°C for 90 min. The cured piece was carefully removed and cut with a razor blade, thoroughly cleaned with tape, sterilized in 70% ethanol and dried in a sterile laminar flow hood. Glass coverslips (ThermoFisher Scientific, Waltham, MA, USA) were immersed in 100% ethanol and flame sterilized in a laminar flow hood. After coating with 0.1 mg/ml poly-D-lysine (Sigma-Aldrich), the chamber was attached on top. Rat cortical neurons were prepared as described above. For each chamber, 2.1x10^5^ neurons were seeded in cortex medium into the ‘soma compartment’. 4 hours later, cells were transduced with AAV vectors (AAV.CTRL: 1.5x10^6^ TU; AAV.ULK1.DN: 4 x10^6^ TU). While maintaining a difference in medium volume between both sides of the chamber to direct axonal growth through the microgrooves, changes of half of the total volume were performed on both sides every to every other day. On DIV 7-9, axons reached the axonal compartment of the chambers. An axotomy was performed by applying air bubbles to the axonal compartment with gentle vacuum aspiration, leading to the induction of axonal degeneration. Hereafter, cortex medium was added again to the axonal compartment.

## Live imaging and quantification of axonal degeneration *in vitro*

For live imaging of axonal degeneration in microfluidic chambers, mCherry-labeled axons were imaged at 37°C and 5% CO_2_ in a microscope incubation system (DMI6000B, Leica, Wetzlar, Germany) equipped with Leica Application Suite software at 16x magnification. Axons were imaged directly before and at 5 min to 6 hours after axotomy. 10 axons were quantified per chamber and only lesioned axons were considered for evaluation. The number of bulb-like structures (diameter > 2 µm) was quantified in single axons within 100-400 µm proximal to the lesion site for all time points in a blinded fashion. The number of bulbs before axotomy was then subtracted from the bulb number at each time point to determine the number of newly formed bulbs^1^.

## Animal experiments

All procedures with AAV.CTRL and AAV.ULK1.DN (stereotactical injection, spinal cord injury, intravitreal virus injection, optic nerve live imaging and crush) were performed under deep anesthesia with 10% ketamine (95 mg/kg body weight) and 2% xylazine (7 mg/kg body weight) injected intraperitoneally. All procedures with the ULK1 inhibitor SBI-0206965 (intravitreal injections, optic nerve live-imaging) were performed under deep anesthesia with intraperitoneal injection of 10% ketamine (75 mg/kg body weight) and 1 mg/ml medetomidine (0.5 mg/kg body weight). To maintain general anesthesia, injections with anesthetics were repeated every 60 min with diminished dosage when necessary. After stereotactical injections and spinal cord injury procedures, animals were given 10 ml of Ringer solution subcutaneously for hydration. Postoperative care was done with metamizole (1.33 mg/ml drinking water) for 3 days. Animals were checked for general health conditions daily.

## Stereotactic viral injection into the red nucleus

In order to express ULK.DN in the red nucleus (RN) neurons and their axons in the rubrospinal tract (RST), stereotactical viral injections into the left RN were performed according to a previously published protocol^12,13^. After anesthesia, animals were placed in a stereotactic frame (David Kopf instruments, Tujunga, CA, USA) and the skull was exposed with a midline incision of the skin. The bregma coordinates for RN injections were: AP -6.0 mm; ML +0.7 mm and DV -6.8 mm. A hole was drilled at the respective coordinates and, using a self-made glass pipette attached to a Nanoliter2000 injector (World Precision Instruments, Sarasota, FL, USA), 2 µl of AAV vectors were injected at a speed of 0.3 µl/min. The exposed skull was closed, and rats were allowed to wake up from anesthesia. Throughout the surgery, the temperature was maintained at 37°C on a heating pad.

## Spinal cord injury

Five weeks after the viral injection into the RN, a spinal cord injury (SCI) was performed as described previously^12,14^. Briefly, the animals were anesthetized as described above, the skin on the back was incised and the fat layer on the back muscles was separated. After splitting of the musculature, the spinal cord was exposed via dorsal laminectomy of the thoracic level 8 (Th8) vertebra. The dorsal right half of the spinal cord was transected at Th8 using a pair of microscissors at a depth of 1.25 mm, which resulted in a complete axotomy of the RST. Finally, back muscles and skin were sutured, and animals allowed to recover from anesthesia. Throughout the surgery, the temperature was maintained at 37°C on a heating pad. The bladder was emptied manually if necessary.

## Spinal cord histology

Seven days after SCI, the animals were killed by CO_2_ insufflation and immediately perfused transcardially with 250 ml phosphate-buffered saline (PBS) followed by 250 ml 4% paraformaldehyde (PFA) in PBS. The spinal cord was dissected, post fixed in 4% PFA ON and dehydrated in gradient sucrose solutions of 10%, 20% and 30%. The samples were then preserved at -20°C until cryosectioning. Spinal cord tissue was partitioned into a block around the lesion (1.5 cm length with the lesion in the center) and a 0.5 cm block at thoracic level 2 (Th2) rostral to the lesion. The lesion block, including the lesion epicenter and the rostral and caudal regions, was cryosectioned horizontally (30 µm) at the level of RST for evaluation of axonal degeneration. Coronal cryosections of the spinal cord at Th2 level were prepared at a thickness of 30 µm for the evaluation of the RST transduction rate. All samples were stored at -20°C until further evaluation.

## Intravitreal injection, optic nerve crush and live-imaging

For the evaluation of acute axonal degeneration *in vivo*, rat optic nerve crush (ONC) and live-imaging were performed as described previously^15^. In brief, the skin of the deeply anesthetized animal was incised close to the orbital rim, the orbital cavity was opened, and the lacrimal gland was moved to the front or partly removed. The superior rectus muscle was detached from its insertion point and the eye bulb was rotated laterally. The optic nerve was exposed by a longitudinal incision of the optic nerve sheath. Care was taken not to damage the central retinal artery. Before the ONC, the rat was transferred to an Axio Examiner.Z1 microscope (Zeiss, Oberkochen, Germany) equipped with a 40x/1.0 NA water immersion objective for live-imaging. Fluorescent images were taken to verify the labeling of the axons and exclude any damage to the nerve. The crush injury was performed by tightly constricting a 10-0 polyamide suture (Johnson & Johnson, New Brunswick, NJ, USA) around the optic nerve at approximately 1 mm from the insertion of the optic nerve into the eye bulb for 30 s. The knot was left on the nerve for visualization of the crush site. Images were taken in the area of 400-500 µm proximal to the crush site at 5-360 min after crush using ZEN software. Images were recorded as Z-stacks with a 0.45 µm interval. Surgical exposure of the area distal to the crush is difficult and imaging of both the area proximal and distal to the lesion site resulted in extensive bleaching and reduced image quality. We therefore limited imaging to the area of 400-500 µm proximal to the lesion site. During the entire procedure, the animal’s body temperature was maintained on a constant level using a warming pad. If necessary, the animal received subcutaneous fluid injections every 2 hours. At the end of the imaging session, animals were killed by insufflation of CO_2_ and cervical dislocation without regaining consciousness.

## Quantification of LC3 and p62 puncta in the optic nerve

Images were taken in visual fields of 78.5 µm x 78.5 µm at approximately 400 µm proximal and distal to the crush lesion. Intraaxonal LC3 and p62 puncta were quantified with ImageJ software. After conversion into 8-bit, the SMI32 image was threshold adjusted to highlight only SMI32 labeled axons. This image was then subtracted from the LC3 or p62 picture using the image calculator. The resulting image contained intraaxonal LC3 or p62 puncta. Intraaxonal puncta in each picture were counted in a blinded fashion and the number of puncta was normalized to the axon area of the image.

# References

1 Zhang JN, Michel U, Lenz C, Friedel CC, Köster S, D’Hedouville Z *et al.* Calpain-mediated cleavage of collapsin response mediator protein-2 drives acute axonal degeneration. *Sci Rep* 2016; **6**: 1–15.

2 Andrews S. FastQC: a quality control tool for high throughput sequence data. 2010.https://www.bioinformatics.babraham.ac.uk/projects/fastqc/ (accessed 14 Dec2018).

3 Dobin A, Davis CA, Schlesinger F, Drenkow J, Zaleski C, Jha S *et al.* STAR: ultrafast universal RNA-seq aligner. *Bioinformatics* 2013; **29**: 15–21.

4 Anders S, Reyes A, Huber W. Detecting differential usage of exons from RNA-seq data. *Genome Res* 2012; **22**: 2008–17.

5 Durinck S, Spellman PT, Birney E, Huber W. Mapping identifiers for the integration of genomic datasets with the R/Bioconductor package biomaRt. *Nat Protoc* 2009; **4**: 1184–91.

6 Liao Y, Smyth GK, Shi W. featureCounts: an efficient general purpose program for assigning sequence reads to genomic features. *Bioinformatics* 2014; **30**: 923–930.

7 Love MI, Huber W, Anders S. Moderated estimation of fold change and dispersion for RNA-seq data with DESeq2. *Genome Biol* 2014; **15**: 550.

8 Zhang Y, Bilbao A, Bruderer T, Luban J, Strambio-De-Castillia C, Lisacek F *et al.* The Use of Variable Q1 Isolation Windows Improves Selectivity in LC-SWATH-MS Acquisition. *J Proteome Res* 2015; **14**: 4359–4371.

9 Lambert JP, Ivosev G, Couzens AL, Larsen B, Taipale M, Lin ZY *et al.* Mapping differential interactomes by affinity purification coupled with data-independent mass spectrometry acquisition. *Nat Methods* 2013; **10**: 1239–1245.

10 Taylor AM, Blurton-Jones M, Rhee SW, Cribbs DH, Cotman CW, Jeon NL. A microfluidic culture platform for CNS axonal injury, regeneration and transport. *Nat Methods* 2005; **2**: 599–605.

11 Park JW, Vahidi B, Taylor AM, Rhee SW, Jeon NL. Microfluidic culture platform for neuroscience research. *Nat Protoc* 2006; **1**: 2128–2136.

12 Challagundla M, Koch JC, Ribas VT, Michel U, Kügler S, Ostendorf T *et al.* AAV-mediated expression of BAG1 and ROCK2-shRNA promote neuronal survival and axonal sprouting in a rat model of rubrospinal tract injury. *J Neurochem* 2015; **134**: 261–275.

13 Ruitenberg MJ, Eggers R, Boer GJ, Verhaagen J. Adeno-associated viral vectors as agents for gene delivery: application in disorders and trauma of the central nervous system. *Methods* 2002; **28**: 182–194.

14 Ribas VT, Schnepf B, Challagundla M, Koch JC, Bähr M, Lingor P. Early and Sustained Activation of Autophagy in Degenerating Axons after Spinal Cord Injury. *Brain Pathol* 2015; **25**: 157–170.

15 Koch J, Knöferle J, Tönges L, Michel U, Bähr M, Lingor P. Imaging of rat optic nerve axons in vivo. *Nat Protoc* 2011; **6**: 1887–96.

# SI FIGURE LEGENDS:

**Supplementary Fig. 1:** **AAV.ULK1.DN does not affect cell survival *in vitro*. (A)** Scheme of experimental setup for toxicity and cell survival assays. DOP: day of preparation of E18 rat cortical neurons. DIV: day in vitro. AAV: transduction with adeno-associated viral vectors. MC: medium change. STA: addition of staurosporine (30-300 nM) 24 hours before toxilight assay. RAP: addition of rapamycin (750 nM) 24 hours before lysis. **(B)** Luminescence relative to non-transduced control measured by a luminometer after treatment with toxilight reagents measures toxicity for different virus titers (3E6-1E8: 3x10^6^-1x10^8^ TU) on DIV8 (n=3 independent cultures). **(C)** Representative immunoblot of caspase 3 with low exposure (to detect the total protein) and high exposure (to detect its cleaved form). **(D-F)** Quantifications of the band intensities of caspase 3 and its cleaved form (cl. casp. 3) normalized to GAPDH as loading control as well as the cl. casp. 3/ total caspase 3 ratio (n=5 independent cultures). **(G)** Luminescence measured by a luminometer after addition of toxilight reagents quantifies cell death after treatment with different concentrations of the apoptosis-inducing agent staurosporine (0-300 nM) on DIV8 (n=3 independent cultures). Data are presented as single data points and means±SEM. *P<0.05, **P<0.01, N.S.: no significant difference according to one-way ANOVA and Sidak’s multiple comparisons test.

**Supplementary Fig. 2: Transduction with AAV.ULK1.DN does not alter the expression of ULK2.** Lysates obtained from E18 rat cortical neurons on DIV 8 after transduction with AAV.CTRL or AAV.ULK1.DN were analyzed by Western blotting. A representative immunoblot of ULK2 is shown on the left, the quantification of band intensities normalized to GAPDH as loading control is depicted on the right (n=5 independent cultures). N.S.: no significant difference according to one-way ANOVA and Sidak’s multiple comparisons test.

**Supplementary Fig. 3: SBI-0206965 increases the number of p62 puncta after optic nerve crush *in vivo*.** **(A)** Scheme of experimental setup. SBI: intravitreal injection of SBI-0206965 or control. ONC: optic nerve crush. IHC: fixation for immunohistochemistry. **(B)** Representative photomicrographs of immunohistochemical staining against the axonal marker SMI-32 (green) and p62 (magenta) in the optic nerve after crush injury and intravitreal injection of SBI or control. Arrows indicate intraaxonal p62 puncta, insets show examples at a higher magnification. Scale bar: 10 µm. **(C)** Quantification of the number of intraaxonal p62 puncta normalized to axon area (20-28 visual fields at approximately 400 µm proximal and distal to lesion, n=5 animals per group). Bars represent single datapoints and means±SEM. *P<0.05 according to one-way ANOVA and Tukey’s multiple comparisons test.

**Supplementary Fig. 4: Analysis of rat spinal cords after injury shows no difference in transduction rate, lesion size and Wallerian degeneration after transduction with AAV.ULK1.DN and AAV.CTRL. (A)** Scheme of experimental setup. AAV: adeno-associated viral vectors. DOI: day of injection of AAV in red nucleus. SCI: spinal cord injury. **(B)** Exemplary coronal sections of the spinal cord at thoracic level 2 after transduction with AAV.CTRL and AAV.ULK1.DN. Scale bar: 500 µm. Inset: higher magnification showing axon labeling in rubrospinal tract. Scale bar: 100 µm. **(C)** Quantification of the total number of labeled axons after transduction with AAV.CTRL and AAV.ULK1.DN (n=5 animals for each viral vector). **(D)** Representative photomicrographs of immunohistochemistry for the Glial fibrillary acidic protein (GFAP, green) in horizontal sections of thoracic spinal cord showing the lesion area after SCI and transduction with AAV.CTRL and AAV.ULK1.DN. Scale bar: 400 µm. **(E)** Quantification of the lesion area after transduction with AAV.CTRL and AAV.ULK1.DN (n=4 animals for each viral vector). **(F)** Exemplary horizontal sections of thoracic spinal cord transduced with control virus and AAV.ULK1.DN depict rubrospinal tract at approximately 4000 µm distal to lesion. mCherry fluorescence is shown in red. Green arrows indicate fragmented axons. Scale bar: 100 µm. **(G)** Quantification of the percentage of degenerated axons caudal to lesion after transduction with AAV.CTRL and AAV.ULK1.DN (n=5 animals for each viral vector). Data are presented as single data points and means±SEM. N.S.: no significant difference according to two-tailed unpaired t-test.

**Supplementary Fig. 5: Overview of the 10 proteins with strongest up- and downregulation in quantitative proteomic profiling after transduction with AAV.ULK1.DN.** Lysates obtained from E18 rat cortical neurons on DIV 8 after transduction with AAV.CTRL or AAV.ULK1.DN were subjected to proteomics analysis. **(A)** Overview of the 10 proteins with strongest up- and downregulation. GGA3 = Golgi Associated, Gamma Adaptin Ear Containing, ARF Binding Protein 3, GCSH = Glycine Cleavage System Protein H, HSBP1 = Heat Shock Factor Binding Protein 1, ACYP2 = Acylphosphatase 2, PDCD10 = Programmed Cell Death Protein 10, SDC3 = Syndecan 3, CTNNA1 = α-E-Catenin, PTRH2 = Peptidyl-tRNA Hydrolase 2, GOLT1B = Golgi Transport 1B, SNRPB = Small Nuclear Ribonucleoprotein Polypeptide B, SUB1 = Activated RNA Polymerase II Transcriptional Coactivator p15, SHROOM2 = Shroom Family Member 2, TAOK1 = Thousand And One Amino Acid Protein Kinase 1, ATXN2 = Ataxin 2, KPNA1 = Importin Subunit Alpha-1, PAK2 = P21 (RAC1) Activated Kinase 2, ELP2 = Elongator Complex Protein 2, KIFAP3 = Kinesin Associated Protein 3, ALDH1L1 = Aldehyde Dehydrogenase 1 Family Member L1, CST3 = Cystatin-C.

**Supplementary Fig. 6: Overview of the genes with significantly differential expression after transduction with AAV.ULK1.DN.** Lysates obtained from E18 rat cortical neurons on DIV 8 after transduction with AAV.CTRL or AAV.ULK1.DN were subjected to differential gene expression analysis (n=3 independent cultures). Only significantly regulated genes (FDR-corrected p-value<0.05) are shown. Ulk1 = unc-51 like autophagy activating kinase 1, Rgs2 = regulator of G-protein signaling 2, Trib3 = tribbles pseudokinase 3, Chac1 = ChaC glutathione-specific gamma-glutamylcyclotransferase 1, Stc2 = stanniocalcin 2, Zfhx4 = zinc finger homeobox 4, Fn1 = fibronectin 1, Fst = follistatin, Tnfrsf1a = TNF receptor superfamily member 1A, F3 = coagulation factor III, tissue factor, Itgb5 = integrin subunit beta 5, Vim = vimentin, Kcnj10 = potassium voltage-gated channel subfamily J member 10, Fbln2 = fibulin 2, Igfbp2 = insulin-like growth factor binding protein 2, Serpinh1 = serpin family H member 1, Pmp22 = peripheral myelin protein 22, Fam181b = family with sequence similarity 181, member B, Tst = thiosulfate sulfurtransferase, Olig2 = oligodendrocyte transcription factor 2, Top2a = DNA topoisomerase II alpha, Prc1 = protein regulator of cytokinesis 1, Tk1 = thymidine kinase 1, Mki67 = marker of proliferation Ki-67, Metrn = meteorin, glial cell differentiation regulator, Aif1l = allograft inflammatory factor 1-like, Paqr8 = progestin and adipoQ receptor family member 8, Slc9a3r1 = SLC9A3 regulator 1, Fgfrl1 = fibroblast growth factor receptor-like 1, Bmp7 = bone morphogenetic protein 7, Pon2 = paraoxonase 2, Efhd1 = EF-hand domain family, member D1, Pdpn = podoplanin, Vcam1 = vascular cell adhesion molecule 1, Nes = nestin, Mdk = midkine, Pld1 = phospholipase D1, Tnc = tenascin C, Gramd2b = GRAM domain containing 2B, Parvb = parvin, beta, Inka2 = family with sequence similarity 212, member B, Acss1 = acyl-CoA synthetase short-chain family member 1, Acsf2 = acyl-CoA synthetase family member 2, Cyp7b1 = cytochrome P450 family 7 subfamily B member 1, Jam2 = junctional adhesion molecule 2, Mmp14 = matrix metallopeptidase 14.

**SUPPLEMENTARY TABLES:**

| **Term** | **Count** | **%** | **P-Value** | **Fold Enrichment** |
| --- | --- | --- | --- | --- |
| GO:0000387~spliceosomal snRNP assembly | 4 | 3.39 | 7.59E-04 | 21.97 |
| GO:0043486~histone exchange | 3 | 2.54 | 0.001438709 | 51.27 |
| GO:0098609~cell-cell adhesion | 6 | 5.08 | 0.011842779 | 4.37 |
| GO:0044210~'de novo' CTP biosynthetic process | 2 | 1.69 | 0.012847345 | 153.82 |
| GO:0042493~response to drug | 9 | 7.63 | 0.020893135 | 2.62 |
| GO:0000398~mRNA splicing, via spliceosome | 4 | 3.39 | 0.025472706 | 6.28 |
| GO:0090168~Golgi reassembly | 2 | 1.69 | 0.025531079 | 76.91 |
| GO:0009052~pentose-phosphate shunt, non-oxidative branch | 2 | 1.69 | 0.031812237 | 61.53 |
| GO:0000395~mRNA 5'-splice site recognition | 2 | 1.69 | 0.038053265 | 51.27 |
| GO:0007049~cell cycle | 4 | 3.39 | 0.042533911 | 5.13 |
| GO:0051683~establishment of Golgi localization | 2 | 1.69 | 0.044254416 | 43.95 |
| GO:0007420~brain development | 6 | 5.08 | 0.046383606 | 3.05 |
| GO:0016192~vesicle-mediated transport | 4 | 3.39 | 0.047969873 | 4.88 |
| GO:0043066~negative regulation of apoptotic process | 8 | 6.78 | 0.04981385 | 2.38 |
| GO:0045454~cell redox homeostasis | 3 | 2.54 | 0.064098477 | 7.21 |
| GO:0006979~response to oxidative stress | 4 | 3.39 | 0.068389859 | 4.21 |
| GO:0006098~pentose-phosphate shunt | 2 | 1.69 | 0.080637949 | 23.66 |
| GO:0046628~positive regulation of insulin receptor signaling pathway | 2 | 1.69 | 0.080637949 | 23.66 |
| GO:0033120~positive regulation of RNA splicing | 2 | 1.69 | 0.086566947 | 21.97 |
| GO:0042542~response to hydrogen peroxide | 3 | 2.54 | 0.090175449 | 5.92 |
| GO:0006754~ATP biosynthetic process | 2 | 1.69 | 0.092458046 | 20.51 |
| GO:2000811~negative regulation of anoikis | 2 | 1.69 | 0.092458046 | 20.51 |
| GO:0043149~stress fiber assembly | 2 | 1.69 | 0.098311484 | 19.23 |
| GO:0009987~cellular process | 2 | 1.69 | 0.098311484 | 19.23 |
| GO:0014070~response to organic cyclic compound | 5 | 4.24 | 0.099094499 | 2.83 |

**Supplementary Table 1: Overview of biological processes annotated to proteins significantly regulated in** **quantitative proteomic profiling after transduction with AAV.ULK1.DN.** Lysates obtained from E18 rat cortical neurons on DIV 8 after transduction with AAV.CTRL or AAV.ULK1.DN were subjected to proteomics analysis. An enrichment analysis of functional annotations in Gene Ontology (GO) was performed for all significantly regulated proteins. The count of annotated proteins, percentage of annotated proteins (%), p-value and fold enrichment value are given for each GO biological process term. Significantly regulated processes (Modified Fisher Exact P-value<0.05) are outlined in orange.

| **Term** | **Count** | | **%** | **P-Value** | **Fold Enrichment** |
| --- | --- | --- | --- | --- | --- |
| GO:0070062~extracellular exosome | | 51 | 43.22 | 2.87E-14 | 3.08 |
| GO:0005737~cytoplasm | | 56 | 47.46 | 6.11E-06 | 1.71 |
| GO:0005685~U1 snRNP | | 5 | 4.24 | 7.78E-06 | 38.01 |
| GO:0005634~nucleus | | 52 | 44.07 | 1.76E-05 | 1.71 |
| GO:0005683~U7 snRNP | | 4 | 3.39 | 1.91E-05 | 70.96 |
| GO:0005913~cell-cell adherens junction | | 10 | 8.47 | 3.45E-05 | 6.16 |
| GO:0005687~U4 snRNP | | 4 | 3.39 | 6.37E-05 | 49.12 |
| GO:0034709~methylosome | | 4 | 3.39 | 8.08E-05 | 45.62 |
| GO:0005739~mitochondrion | | 24 | 20.34 | 1.15E-04 | 2.39 |
| GO:0034719~SMN-Sm protein complex | | 4 | 3.39 | 1.78E-04 | 35.48 |
| GO:0005682~U5 snRNP | | 4 | 3.39 | 2.10E-04 | 33.61 |
| GO:0005686~U2 snRNP | | 4 | 3.39 | 3.77E-04 | 27.77 |
| GO:0005689~U12-type spliceosomal complex | | 4 | 3.39 | 6.82E-04 | 22.81 |
| GO:0043209~myelin sheath | | 7 | 5.93 | 0.00123469 | 5.85 |
| GO:0005829~cytosol | | 21 | 17.80 | 0.001785607 | 2.11 |
| GO:0005654~nucleoplasm | | 22 | 18.64 | 0.002315532 | 2.01 |
| GO:0071013~catalytic step 2 spliceosome | | 5 | 4.24 | 0.002909345 | 8.40 |
| GO:0005925~focal adhesion | | 9 | 7.63 | 0.003299353 | 3.63 |
| GO:0016020~membrane | | 25 | 21.19 | 0.004046228 | 1.81 |
| GO:0071004~U2-type prespliceosome | | 3 | 2.54 | 0.004891202 | 28.17 |
| GO:0005794~Golgi apparatus | | 13 | 11.02 | 0.005343389 | 2.51 |
| GO:0030529~intracellular ribonucleoprotein complex | | 5 | 4.24 | 0.006670896 | 6.65 |
| GO:0048471~perinuclear region of cytoplasm | | 11 | 9.32 | 0.006671261 | 2.74 |
| GO:0005697~telomerase holoenzyme complex | | 3 | 2.54 | 0.008141574 | 21.77 |
| GO:0016607~nuclear speck | | 5 | 4.24 | 0.012172845 | 5.58 |
| GO:0005681~spliceosomal complex | | 4 | 3.39 | 0.012660827 | 8.19 |
| GO:0005743~mitochondrial inner membrane | | 7 | 5.93 | 0.01345952 | 3.57 |
| GO:0043234~protein complex | | 10 | 8.47 | 0.01390254 | 2.61 |
| GO:0071011~precatalytic spliceosome | | 3 | 2.54 | 0.016791188 | 14.97 |
| GO:0042995~cell projection | | 4 | 3.39 | 0.021951496 | 6.65 |
| GO:0044297~cell body | | 4 | 3.39 | 0.022551973 | 6.58 |
| GO:0030864~cortical actin cytoskeleton | | 3 | 2.54 | 0.030521683 | 10.89 |
| GO:0043005~neuron projection | | 7 | 5.93 | 0.035947002 | 2.84 |
| GO:0097526~spliceosomal tri-snRNP complex | | 2 | 1.69 | 0.042671918 | 45.62 |
| GO:0034715~pICln-Sm protein complex | | 2 | 1.69 | 0.042671918 | 45.62 |
| GO:0000243~commitment complex | | 2 | 1.69 | 0.042671918 | 45.62 |
| GO:0000790~nuclear chromatin | | 5 | 4.24 | 0.045119862 | 3.71 |
| GO:0016342~catenin complex | | 2 | 1.69 | 0.054528856 | 35.48 |
| GO:0005789~endoplasmic reticulum membrane | | 8 | 6.78 | 0.058216972 | 2.30 |
| GO:0005802~trans-Golgi network | | 4 | 3.39 | 0.072181287 | 4.12 |
| GO:0022625~cytosolic large ribosomal subunit | | 4 | 3.39 | 0.091801362 | 3.71 |
|  | |  |  |  |  |

**Supplementary Table 2: Overview of cellular components annotated to proteins significantly regulated in** **quantitative proteomic profiling after transduction with AAV.ULK1.DN.** Lysates obtained from E18 rat cortical neurons on DIV 8 after transduction with AAV.CTRL or AAV.ULK1.DN were subjected to proteomics analysis. An enrichment analysis of functional annotations in Gene Ontology (GO) was performed for all significantly regulated proteins. The count of annotated proteins, percentage of annotated proteins (%), p-value and fold enrichment value are given for each GO cellular component term. Significantly regulated GO terms (Modified Fisher Exact P-value<0.05) are outlined in orange.

| **Term** | **Count** | **%** | **P-Value** | **Fold Enrichment** | |
| --- | --- | --- | --- | --- | --- |
| GO:0043525~positive regulation of neuron apoptotic process | 3 | 8.33 | 0.007266673 | | 22.63 |
| GO:0047497~mitochondrion transport along microtubule | 2 | 5.56 | 0.010222829 | | 188.55 |
| GO:0085029~extracellular matrix assembly | 2 | 5.56 | 0.011916783 | | 161.61 |
| GO:0050731~positive regulation of peptidyl-tyrosine phosphorylation | 3 | 8.33 | 0.013109104 | | 16.64 |
| GO:0031102~neuron projection regeneration | 2 | 5.56 | 0.015296287 | | 125.70 |
| GO:0007409~axonogenesis | 3 | 8.33 | 0.016999073 | | 14.50 |
| GO:0001764~neuron migration | 3 | 8.33 | 0.018962936 | | 13.68 |
| GO:0008285~negative regulation of cell proliferation | 4 | 11.11 | 0.025528533 | | 6.05 |
| GO:0030182~neuron differentiation | 3 | 8.33 | 0.029412689 | | 10.81 |
| GO:0043547~positive regulation of GTPase activity | 4 | 11.11 | 0.029950269 | | 5.68 |
| GO:0046777~protein autophosphorylation | 3 | 8.33 | 0.040086141 | | 9.12 |
| GO:0048675~axon extension | 2 | 5.56 | 0.043574474 | | 43.51 |
| GO:0007274~neuromuscular synaptic transmission | 2 | 5.56 | 0.05011318 | | 37.71 |
| GO:1901216~positive regulation of neuron death | 2 | 5.56 | 0.05011318 | | 37.71 |
| GO:0007528~neuromuscular junction development | 2 | 5.56 | 0.059840284 | | 31.42 |
| GO:0048709~oligodendrocyte differentiation | 2 | 5.56 | 0.069471067 | | 26.94 |
| GO:0010506~regulation of autophagy | 2 | 5.56 | 0.074250626 | | 25.14 |
| GO:0042594~response to starvation | 2 | 5.56 | 0.086880387 | | 21.35 |
| GO:0007265~Ras protein signal transduction | 2 | 5.56 | 0.094689232 | | 19.51 |

**Supplementary Table 3: Overview of biological processes annotated to genes with significant differential exon expression after transduction with AAV.ULK1.DN.** Lysates obtained from E18 rat cortical neurons on DIV 8 after transduction with AAV.CTRL or AAV.ULK1.DN were subjected to differential exon expression analysis. An enrichment analysis of functional annotations in Gene Ontology (GO) was performed for all genes with significant differential exon expression. The count of annotated genes, percentage of annotated genes (%), p-value and fold enrichment value are given for each GO biological process term. Significantly regulated processes (Modified Fisher Exact P-value<0.05) are outlined in orange.

| **Term** | **Count** | **%** | **P-Value** | **Fold Enrichment** |
| --- | --- | --- | --- | --- |
| GO:0008017~microtubule binding | 5 | 13.89 | 3.44E-04 | 14.16 |
| GO:0005515~protein binding | 9 | 25.00 | 0.002866453 | 3.37 |
| GO:0005509~calcium ion binding | 5 | 13.89 | 0.024724913 | 4.27 |
| GO:0016887~ATPase activity | 3 | 8.33 | 0.03348841 | 10.02 |
| GO:0031593~polyubiquitin binding | 2 | 5.56 | 0.042905934 | 44.05 |
| GO:0051879~Hsp90 protein binding | 2 | 5.56 | 0.053736955 | 34.98 |
| GO:0048365~Rac GTPase binding | 2 | 5.56 | 0.059873015 | 31.30 |
| GO:0005524~ATP binding | 6 | 16.67 | 0.071890511 | 2.54 |
| GO:0030971~receptor tyrosine kinase binding | 2 | 5.56 | 0.079551717 | 23.32 |

**Supplementary Table 4: Overview of molecular functions annotated to genes with significant differential exon expression after transduction with AAV.ULK1.DN.** . Lysates obtained from E18 rat cortical neurons on DIV 8 after transduction with AAV.CTRL or AAV.ULK1.DN were subjected to differential exon expression analysis. An enrichment analysis of functional annotations in Gene Ontology (GO) was performed for all genes with significant differential exon expression. The count of annotated genes, percentage of annotated genes (%), p-value and fold enrichment value are given for each GO molecular function term. Significantly regulated GO terms (Modified Fisher Exact P-value<0.05) are outlined in orange.

| **Term** | **Count** | **%** | **P-Value** | **Fold Enrichment** |
| --- | --- | --- | --- | --- |
| GO:0005737~cytoplasm | 19 | 52.78 | 0.002045852 | 1.92 |
| GO:0030426~growth cone | 4 | 11.11 | 0.002495712 | 14.30 |
| GO:0005874~microtubule | 4 | 11.11 | 0.009137822 | 8.97 |
| GO:0030122~AP-2 adaptor complex | 2 | 5.56 | 0.01278247 | 151.18 |
| GO:0043025~neuronal cell body | 5 | 13.89 | 0.016587416 | 4.90 |
| GO:0043005~neuron projection | 4 | 11.11 | 0.034920727 | 5.39 |
| GO:0005875~microtubule associated complex | 2 | 5.56 | 0.04492779 | 42.33 |
| GO:0044295~axonal growth cone | 2 | 5.56 | 0.055415359 | 34.14 |
| GO:0060076~excitatory synapse | 2 | 5.56 | 0.064069539 | 29.40 |
| GO:0014069~postsynaptic density | 3 | 8.33 | 0.070532359 | 6.67 |
| GO:0005768~endosome | 3 | 8.33 | 0.076799024 | 6.35 |
| GO:0005905~clathrin-coated pit | 2 | 5.56 | 0.079453526 | 23.52 |
| GO:0030136~clathrin-coated vesicle | 2 | 5.56 | 0.089572865 | 20.75 |
| GO:0005871~kinesin complex | 2 | 5.56 | 0.091248891 | 20.35 |

**Supplementary Table 5: Overview of cellular components annotated to genes with significant differential exon expression after transduction with AAV.ULK1.DN.** . Lysates obtained from E18 rat cortical neurons on DIV 8 after transduction with AAV.CTRL or AAV.ULK1.DN were subjected to differential exon expression analysis. An enrichment analysis of functional annotations in Gene Ontology (GO) was performed for all genes with significant differential exon expression. The count of annotated genes, percentage of annotated genes (%), p-value and fold enrichment value are given for each GO cellular component term. Significantly regulated GO terms (Modified Fisher Exact P-value<0.05) are outlined in orange.
