# Supplementary figures and images for: Inhibition of the autophagic protein ULK1 attenuates axonal degeneration in vitro and in vivo, enhances translation, and modulates splicing
Source: Cell Death Differ. 2020 Apr 27;27(10):2810–27. doi: 10.1038/s41418-020-0543-y (PMC7493890; doi:10.1038/s41418-020-0543-y)

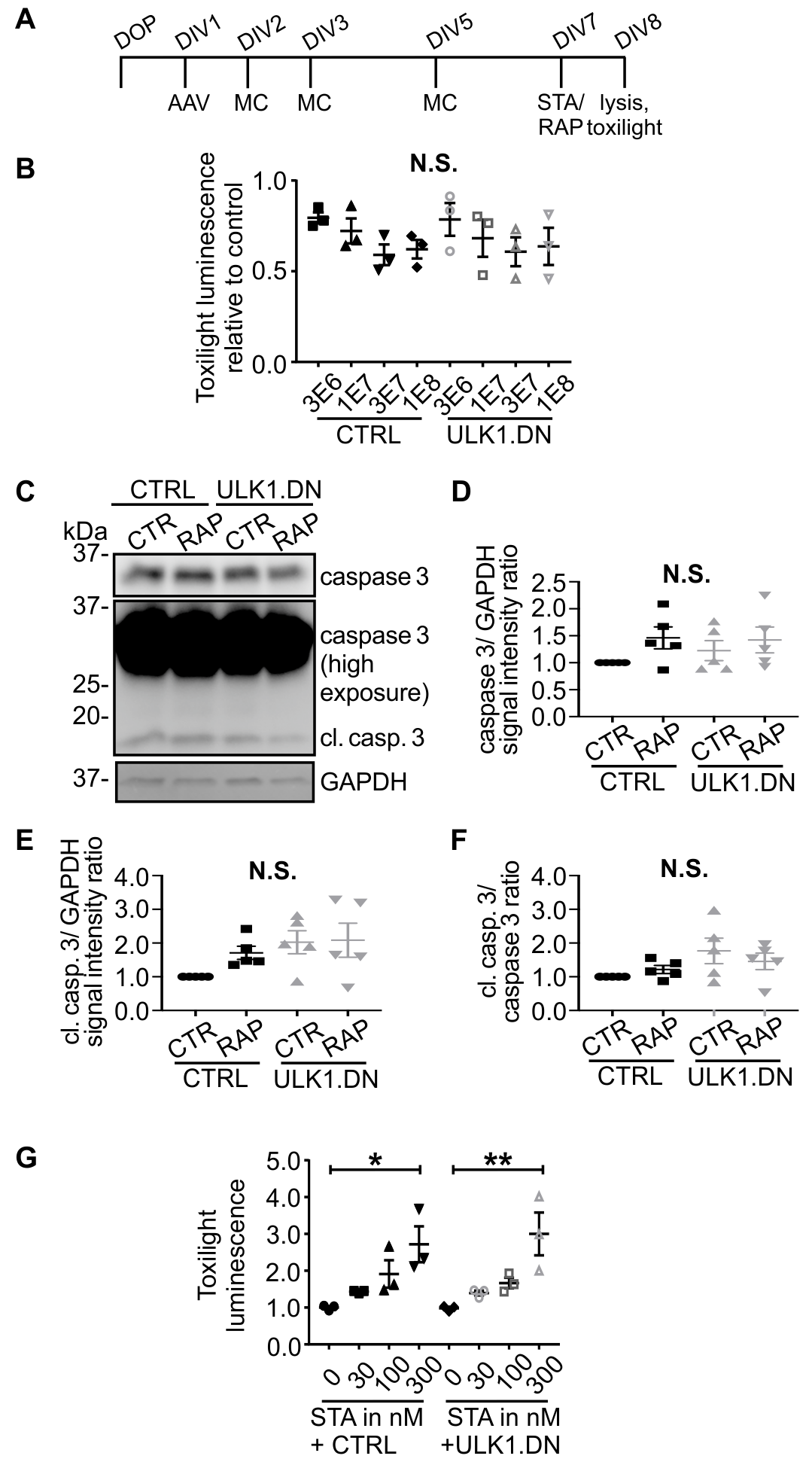

Supplement: Supplementary file 2 — Supplementary Figure S1 [file 41418_2020_543_MOESM2_ESM.tif]

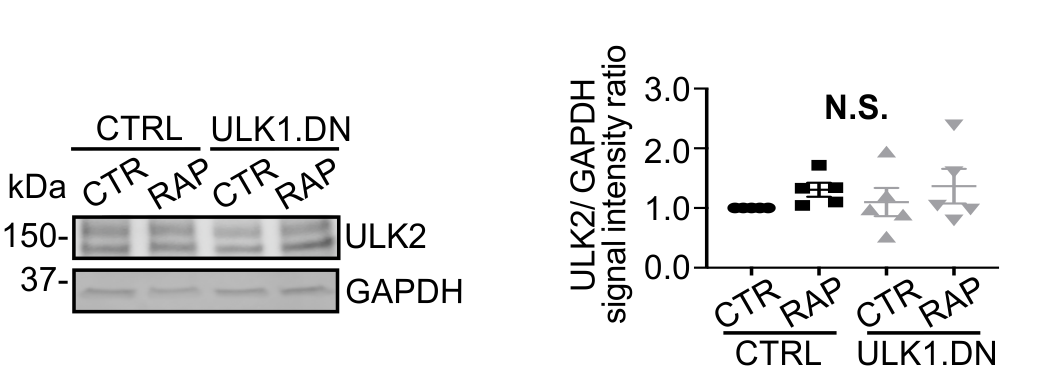

Supplement: Supplementary file 3 — Supplementary Figure S2 [file 41418_2020_543_MOESM3_ESM.tif]

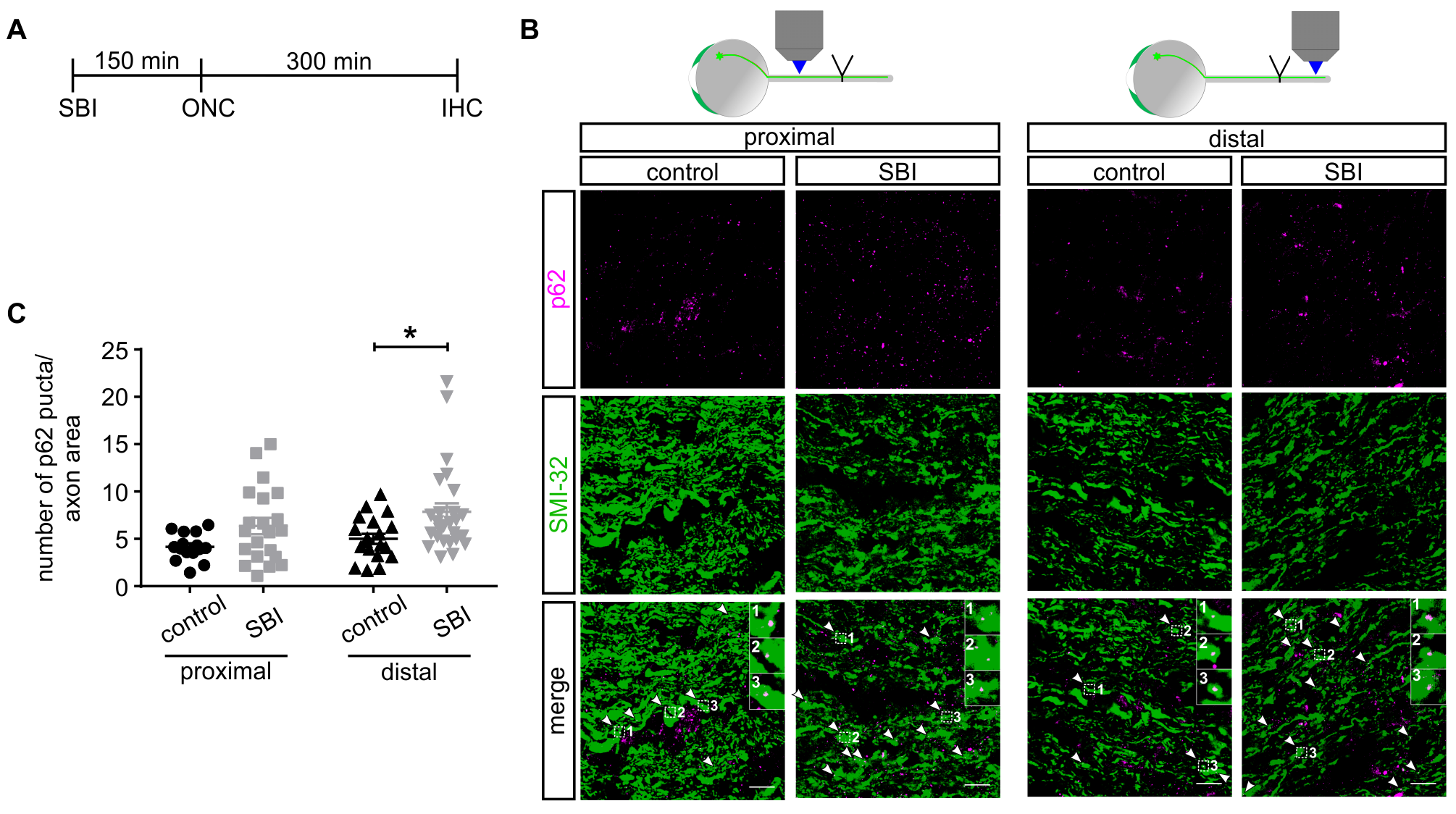

Supplement: Supplementary file 4 — Supplementary Figure S3 [file 41418_2020_543_MOESM4_ESM.tif]

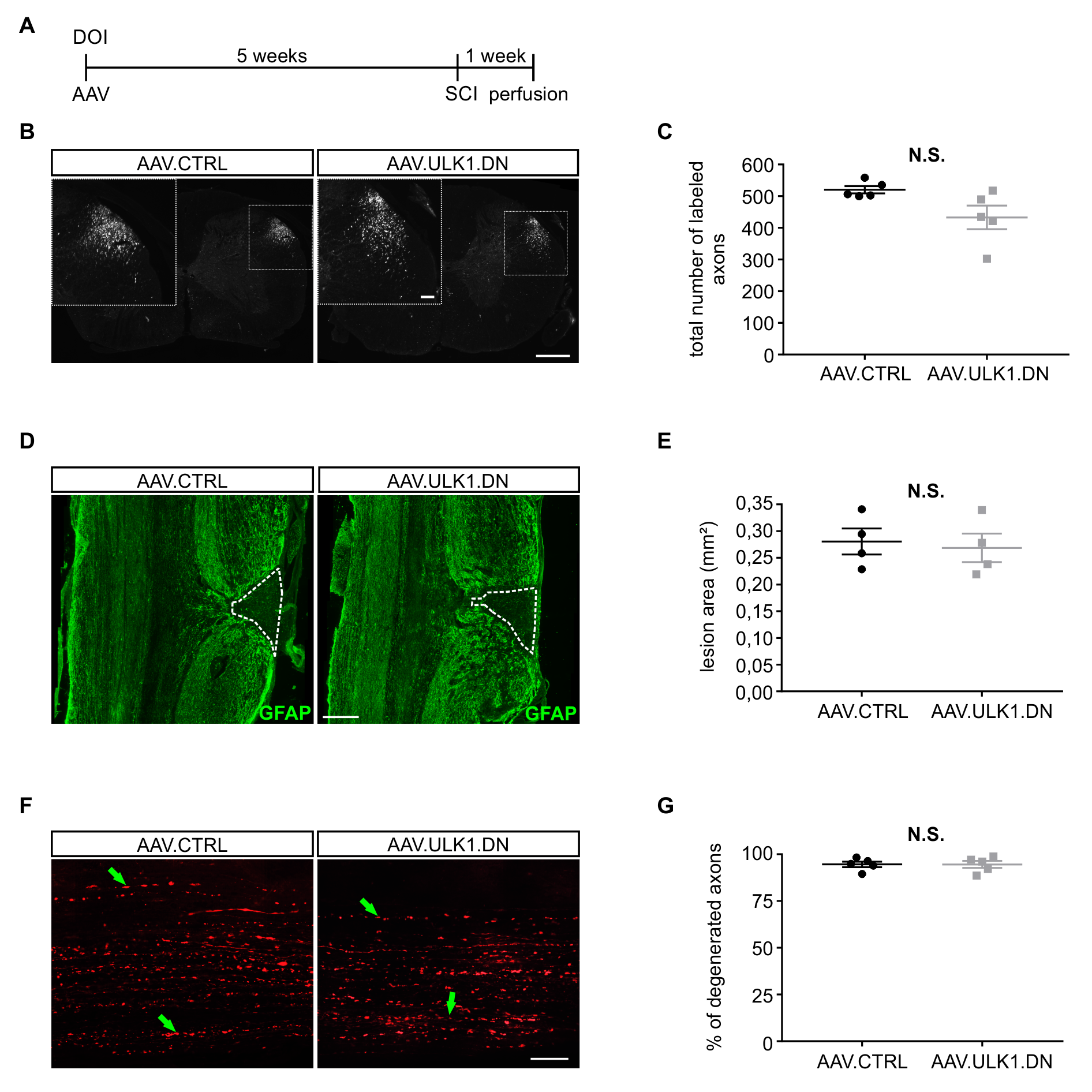

Supplement: Supplementary file 5 — Supplementary Figure S4 [file 41418_2020_543_MOESM5_ESM.tif]

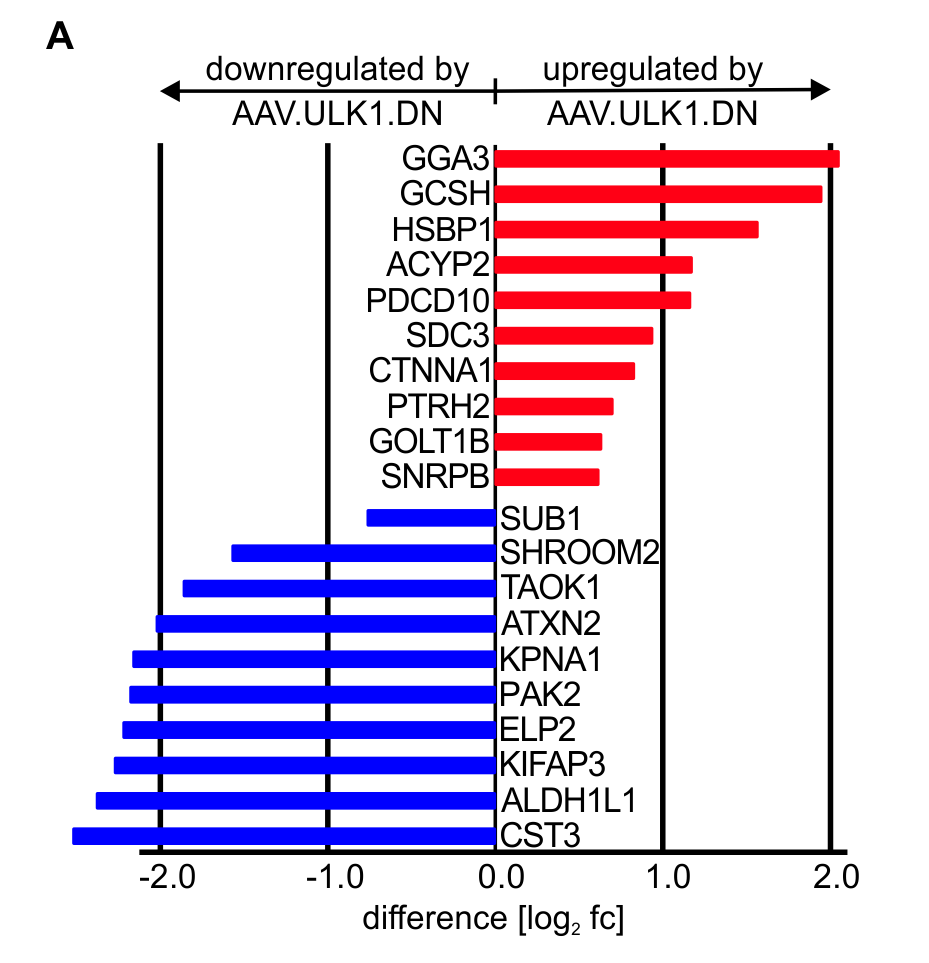

Supplement: Supplementary file 6 — Supplementary Figure S5 [file 41418_2020_543_MOESM6_ESM.tif]

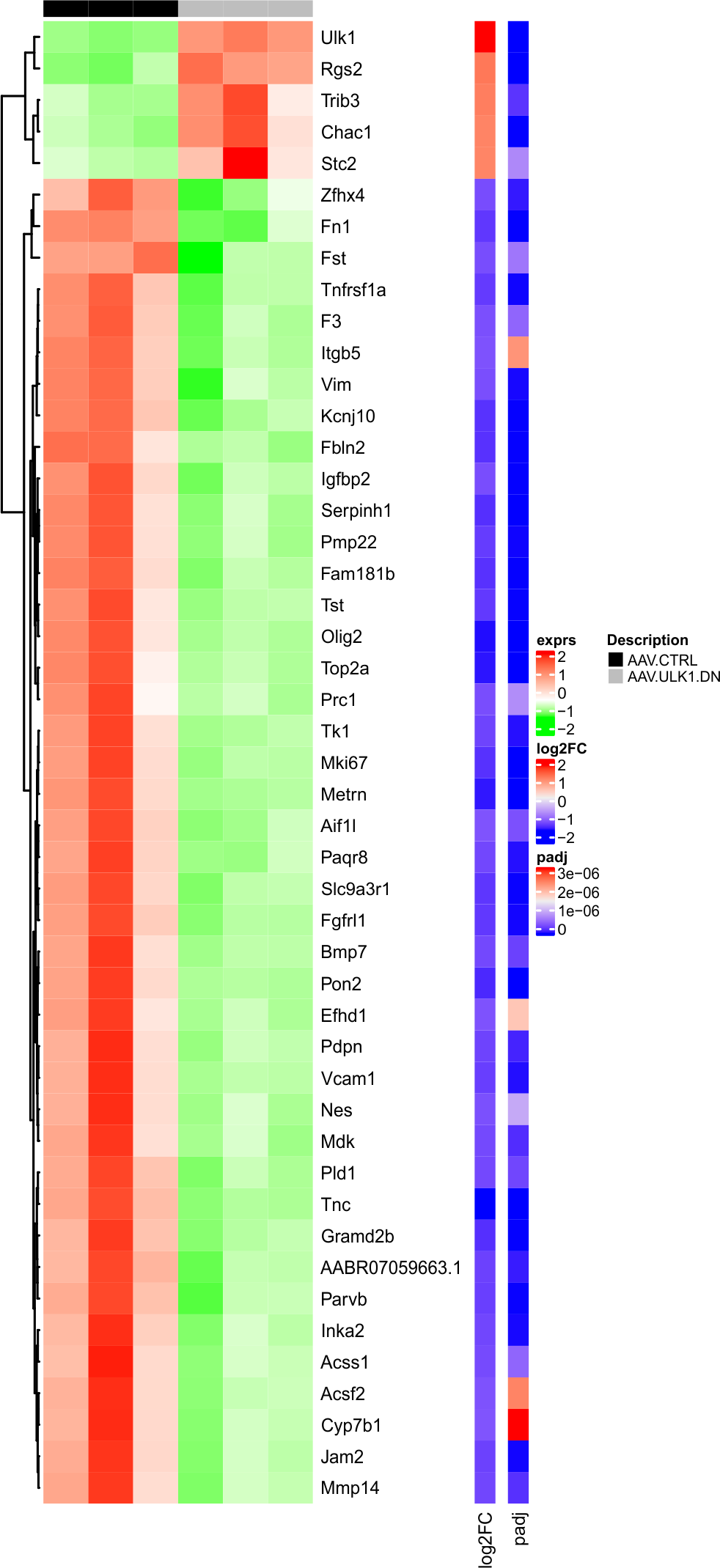

Supplement: Supplementary file 7 — Supplementary Figure S6 [file 41418_2020_543_MOESM7_ESM.tif]
